# Supplementary material for: Single-molecule imaging reveals DNA shape read-out by the INO80 chromatin remodeler
Source: Nat Commun. 2026 Sep 29;17:10320. doi: 10.1038/s41467-026-77906-1 (PMC13623949; doi:10.1038/s41467-026-77906-1)
Supplement: Supplementary file 1 [file 41467_2026_77906_MOESM1_ESM.pdf]

Supplementary Information

Single-Molecule Imaging Reveals DNA Shape Read-out by the INO80 Chromatin Remodeler

Garp Linder, Daniela Horky, Sarah Zernia, and Johannes Stigler*

* To whom correspondence should be addressed: J. Stigler (johannes.stigler@lmu.de)

I. SUPPLEMENTARY NOTES

A. Supplementary Note 1: Calculation of the free-energy barrier

The diffusive motion of a protein along DNA in solution is retarded by two factors: the friction it experiences while moving through the solution and the interactions between DNA and protein. The amount of friction the protein experiences depends on its size, as measured by its radius R. Depending on the nature of its DNA-binding, a protein can diffuse by moving linearly over the DNA employing a rotation-decoupled mechanism [1, 2] or by tracking along the helical axis in a rotational fashion [3]. The diffusion coefficient D scales with $1/R$ for a protein that moves linearly along the DNA and with $1/R^3$ if it additionally rotates along the DNA helix. The second retarding factor is the sum of the protein's interactions with the DNA. To move, it must constantly form and break bonds with DNA, giving rise to free energy barriers along the pathway of diffusion. The height of these energy barriers depends on the characteristics of the protein-DNA contacts and is further modified by the DNA sequence and shape. The resulting retardation is represented by the term $F(\varepsilon) = e^{-\left(\frac{\varepsilon}{k_{\mathrm{B}}T}\right)^2}$, where ε denotes the root-mean-squared variation of the different free-energy barriers that the protein encountered during diffusion (fig. 2B). Altogether, the diffusive motion of the protein can then be described by:

$$D = \frac{k_{\mathrm{B}}T}{\gamma_{\mathrm{lin}} + \gamma_{\mathrm{rot}}} F(\varepsilon) = \frac{k_{\mathrm{B}}T}{6\pi\eta R + \left(\frac{2\pi}{b}\right)^2 \left(8\pi\eta R^3 + 6\pi\eta R R_{\mathrm{OC}}^2\right)} e^{-\left(\frac{\varepsilon}{k_{\mathrm{B}}T}\right)^2} \tag{S1}$$

where γ_{lin} and γ_{rot} represent linear and rotational drag, η is the solvent viscosity, b is the distance between base pairs and R_{OC} is the distance of the protein from the DNA [3]. For proteins moving linearly along DNA γ_{rot} is zero. Models predict ε values between 0.5 to 2 $k_{\mathrm{B}}T$ for efficient target site search [4], which has been confirmed for a wide variety of DBPs [3, 5]. INO80 could hypothetically move along the DNA via a linear or a rotationally coupled mechanism. By measuring D and estimating R as well as R_{OC} from cryo-EM structures, we calculate ε for both possibilities and compare which value is in agreement with the models. Additionally, as explained above, the ε value can give insights into the nature of protein-DNA contacts that occur during binding. Indeed, it has been shown that proteins that induce large conformational changes into the DNA upon binding, e.g., strong bending of the helical axis, exhibit high ε values between 1.4 to 2 $k_{\mathrm{B}}T$.

B. Supplementary Note 2: Kaplan-Meier curves

In fig. S1D, fig. 5D and fig. S6B we plot INO80 lifetime and nucleosome interaction data in form of a survival curve using the Kaplan–Meier method [6]. In our case, it shows the cumulative survival probabilities of fluorophores (fig. S1D) or interactions (fig. 5D and fig. S6B) at time t and is described by:

$$\hat{S}(t) = \prod_{i:t_i \leq t} \left(1 - \frac{d_i}{n_i}\right) \tag{S2}$$

where d_i is the number of bleached fluorophores or dissolved interactions and d_i the number of fluorophores/interactions at risk. It allows inclusion of fluorophores or interactions that did not bleach or dissolve within the observed time interval (censored data) and can be fitted to extract single or multiple lifetimes (τ_1, τ_2...), depending on the complexity of the interaction.

II. SUPPLEMENTARY FIGURES

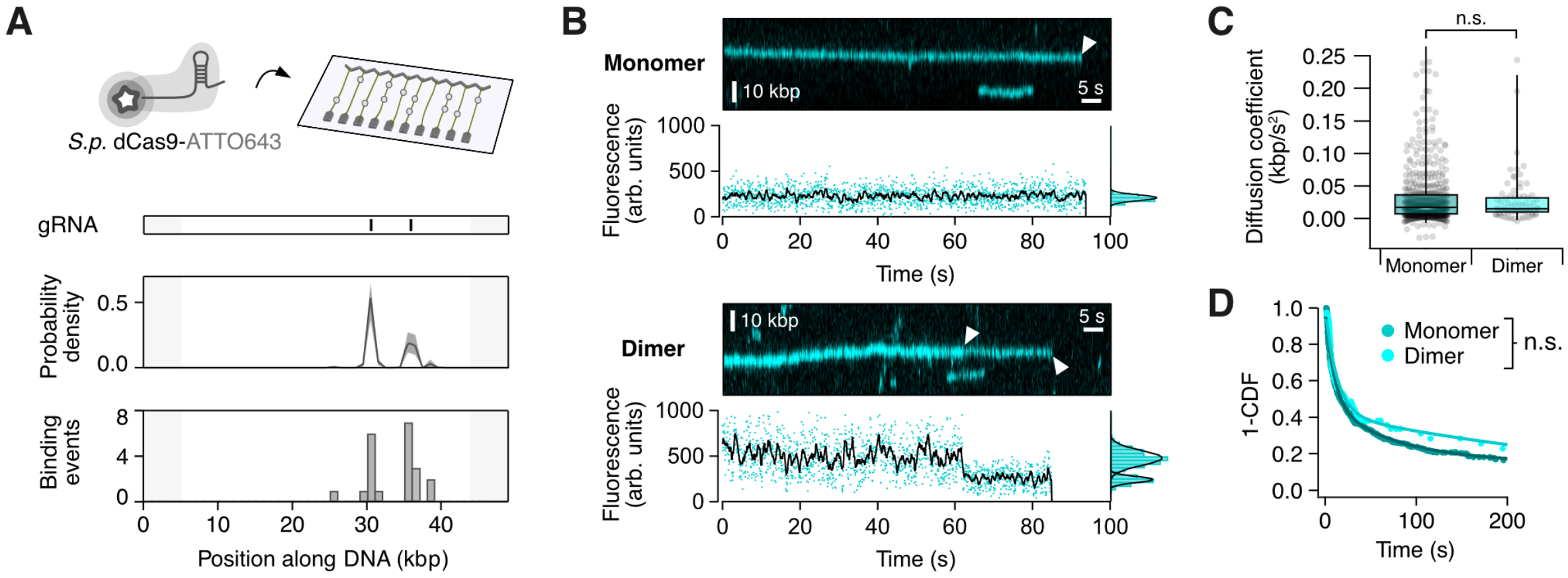

FIG. S1. Photobleaching of monomeric and dimeric INO80 complexes. **(A)** Top: Schematic of dCas9 and DNA curtains assay. gRNA-binding motifs, probability density of localizations and binding events of dCas9 along λ-DNA. Data are presented as mean values +/- 68 % intervals from bootstrapping. $n = 21$ molecules. **(B)** Example kymograms and fluorescence intensities of monomeric (top) and dimeric (bottom) INO80 complexes. **(C)** Comparison of diffusion coefficients of INO80 on λ-DNA for different multimeric states. Box plot shows the median, 25 % and 75 % quartiles and 10th and 90th percentiles. Significance was determined using a Mann-Whitney U test. $n_{\mathrm{Monomer}} = 604$ molecules. $n_{\mathrm{Dimer}} = 73$. **(D)** Survival plot of INO80 on λ-DNA for different multimeric states. Rates were compared with Log-Rank test. n.s. = Not significant. Source data are provided as a Source Data file.

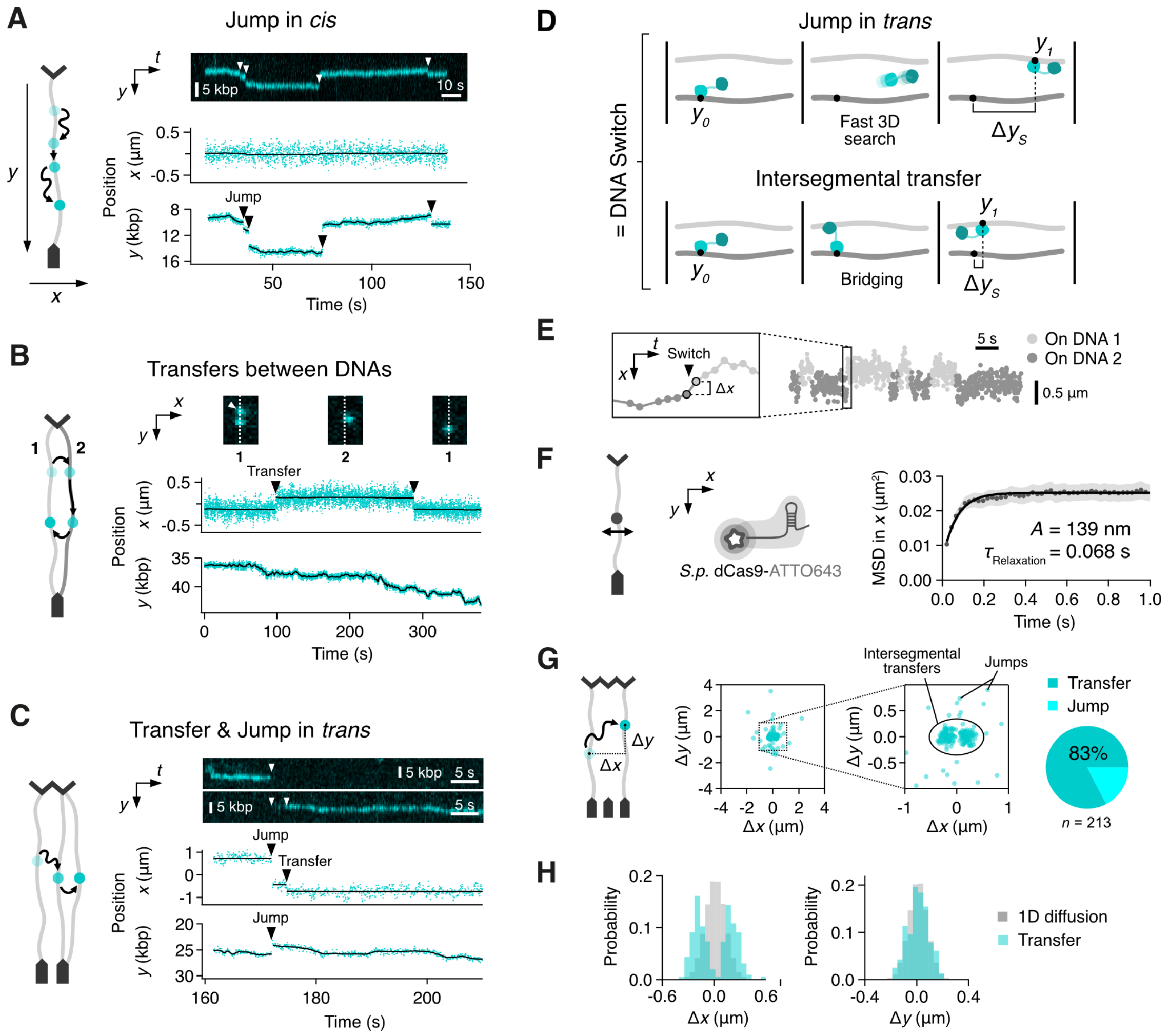

FIG. S2. Intersegmental transfers and jumps of INO80 complexes. **(A)** Example of four long-distance jumps of a single INO80 complex along the same DNA strand. Left: Schematic of the jumping events on the same DNA. Right top: Kymogram of the trajectory. Triangles indicate jumps. Right bottom: x- and y-position of the complex over time. **(B)** Example of two transfer events by a single INO80 complex. Left: Schematic of the transfer events from DNA (1) to (2) to (1). Right top: TIRF microscopy images of the complex before and after each transfer event (striped line represents position of DNA (1)). White triangles indicates relevant INO80 complex. Right bottom: x- and y-position of the complex over time. **(C)** Example of a jumping event between two DNAs followed by a transfer event. Rest as fig. S2A. **(D)** Schematic of DNA switch mechanisms structurally available to the INO80 complex. Switching using "jumps" entails complete dissociation of all DNA-binding domains and an intermediate 3D search; "intersegmental transfers" are performed through a bridging intermediate in which each DNA is bound by a different DNA-binding interface of the complex. Due to mechanistic restrictions, transfers are, on average, accompanied by a much smaller instantaneous change in y- or x-position. **(E)** Example x-trajectory of an INO80 complex. Approximate switch timeframes were identified using an arHMM and localizations were assigned to one of two DNAs. **(F)** Schematic depicting transversal movement of the λ-DNA in x, measured using a dCas9-marker protein. Right: Average MSD in x-direction of dCas9-markers, fitted amplitude and relaxation time of the transversal DNA-movement. $n = 21$ molecules. **(G)** Left: Schematic depicting a jump of an INO80 molecule and the according x- and y-displacements. Middle: Displacements in x and y for inter-DNA switches and jumps identified either by an arHMM or inter-trajectory jump analysis. Right: Middle plot zoomed in towards the origin. $n = 213$ switches/jumps. **(H)** Analysis of intersegmental transfers. Displacements within the two clusters (cyan) were compared to INO80 displacements during 1D search (grey). Left: Histogram of x-displacements. Right: Histogram of y-displacements. $n = 176$ transfers. Source data are provided as a Source Data file.

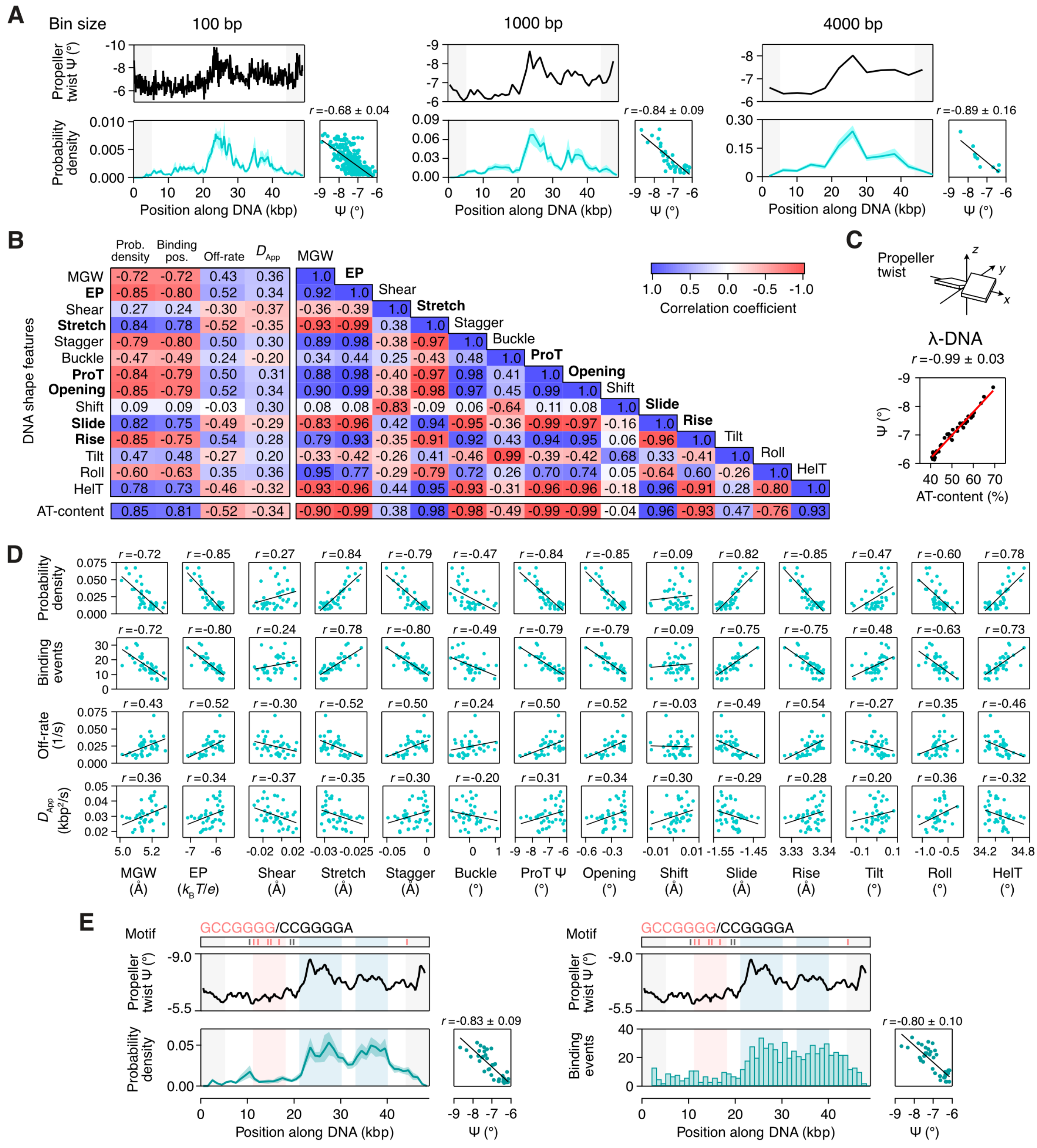

FIG. S3. INO80 shape correlations. **(A)** Effect of bin size on correlations of INO80 probability density with mean propeller twist values across the λ-DNA. Data are presented as mean values +/- 68 % intervals from bootstrapping. **(B)** Pearson's correlation coefficients for correlation of different DNA shape features and AT-content with DNA binding parameters of INO80 along λ-DNA shown in fig. 2F and for correlation among themselves. **(C)** Top: Scheme showing propeller twisting of a DNA basepair. Bottom: Correlation of average propeller twist and AT-content of the λ-DNA used in experiments (1 kbp window). **(D)** Full correlation plots and Pearson's correlation coefficients of different DNA shape features with DNA binding parameters of INO80 along λ-DNA shown in fig. S3B. **(E)** Nhp10-Motifs, average propeller twist (1 kb sliding window), probability density (left) and binding events (right) of INO80 along λ-DNA at 120 mM KGlu without ATP. Right boxes display correlation of binding parameters with average propeller twist and Pearson's correlation coefficient. Shaded areas show chrome barriers (gray), Nhp10 motifs (red) and areas of high negative propeller twist (blue). Grey shaded areas were not included in the correlation analysis. Data are presented as mean values +/- 68 % intervals from bootstrapping. $n = 719$ molecules. Source data are provided as a Source Data file.

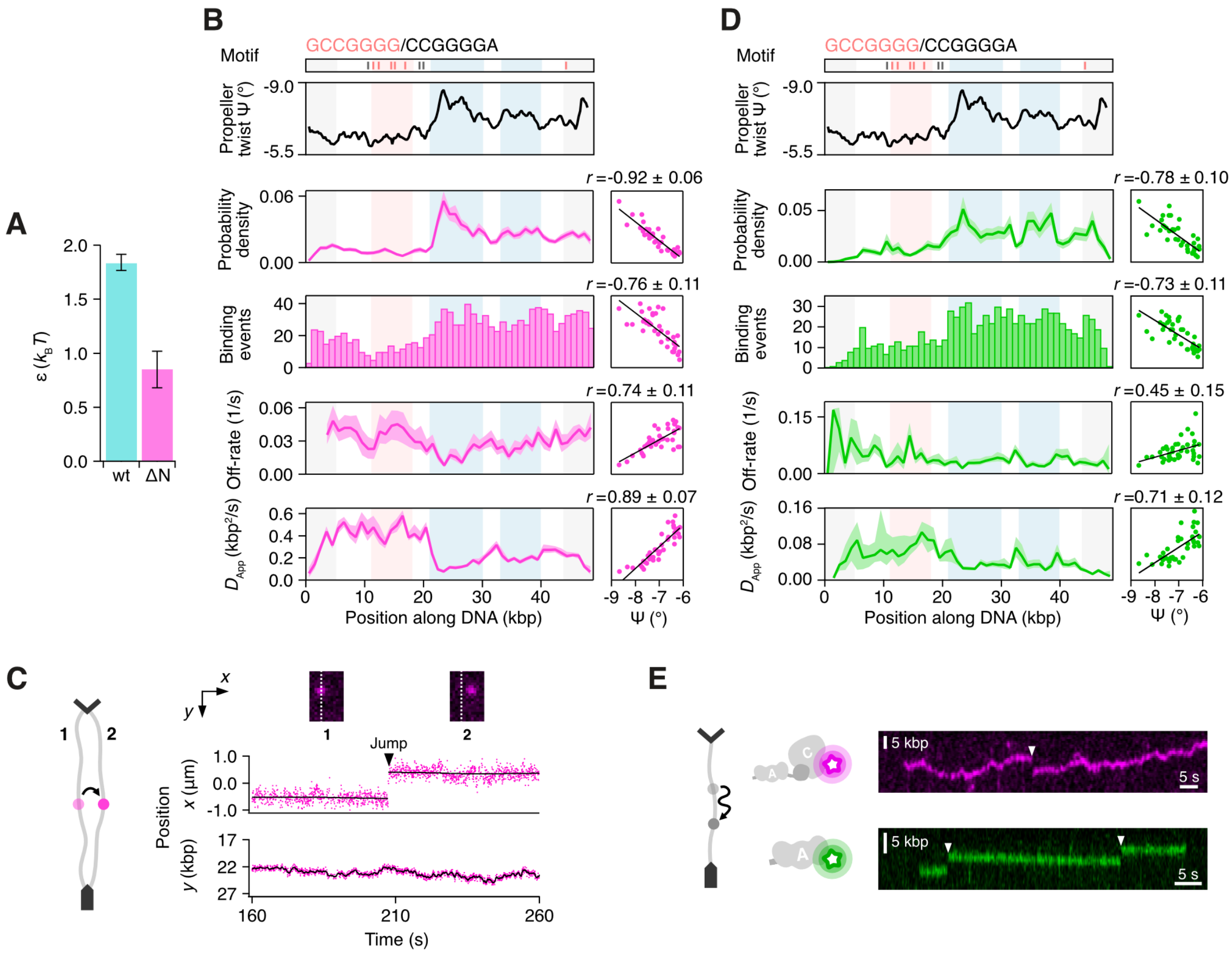

FIG. S4. The A-module is a DNA shape sensor. **(A)** Comparison of free-energy barrier ε for *Sc.* wildtype and *Ct.* ΔN-mutant of INO80. Error bars indicate uncertainty from the range of estimated hydrodynamic radii R for both complexes. **(B)** Nhp10-Motifs, average propeller twist (1 kb sliding window) and different binding parameters of *Ct.* ΔN-INO80 along λ-DNA. The right boxes display the correlation between binding parameters and the average propeller twist, as well as Pearson's correlation coefficient. Shaded areas show chrome barriers (gray), Nhp10 motifs (red) and areas of high negative propeller twist (blue). Grey shaded areas were not included in the correlation analysis. Data are presented as mean values +/- 68 % intervals from bootstrapping. $n = 1180$ molecules. **(C)** As fig. S2B, but for *Ct.* ΔN-INO80. **(D)** As fig. S4B, but for the A-module. $n = 851$ molecules. **(E)** Example of long-distance jumps in *cis* of single mutant complexes along the same DNA strand for *Ct.* ΔN-INO80 and A-module. White triangles indicate jumps. Source data are provided as a Source Data file.

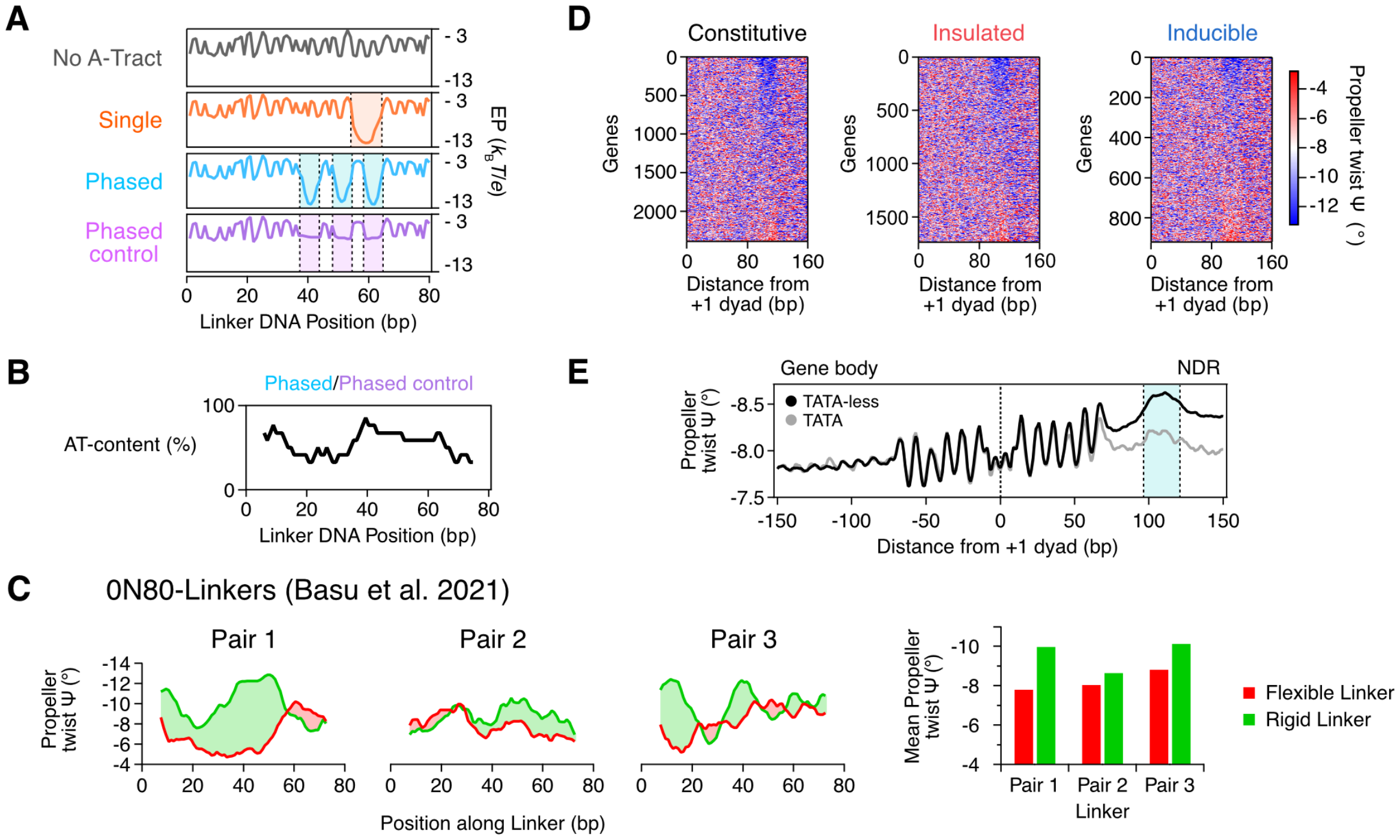

FIG. S5. Effect of DNA shape sensing on nucleosome remodeling. **(A)** Predicted EP for linker DNA of all nucleosome constructs. Areas highlighted indicate changes in shape compared to the "No A-Tract" sequence. **(B)** AT-content for linker DNA of "phased" and "phased control" constructs (10 bp sliding window). **(C)** DNA Shape analysis of 0N80-Linkers used in [7]. Left: Average propeller twist (15 bp sliding window) along the linker sequence for all three linker pairs tested. Right: Average propeller twist for all linker sequences compared. **(D)** Shape profiles around +1 nucleosomes of respective promoter class, sorted by average propeller twist between −95 to −120 bp from +1 nucleosome dyad. **(E)** Average shape profiles around +1-nucleosomes of TATA and TATA-less genes in *S. cerevisiae* oriented according to the direction of transcription. The region of high propeller twist is highlighted. $n_{\text{TATA-less}} = 4528$ promoter. $n_{\text{TATA}} = 1014$. Source data are provided as a Source Data file.

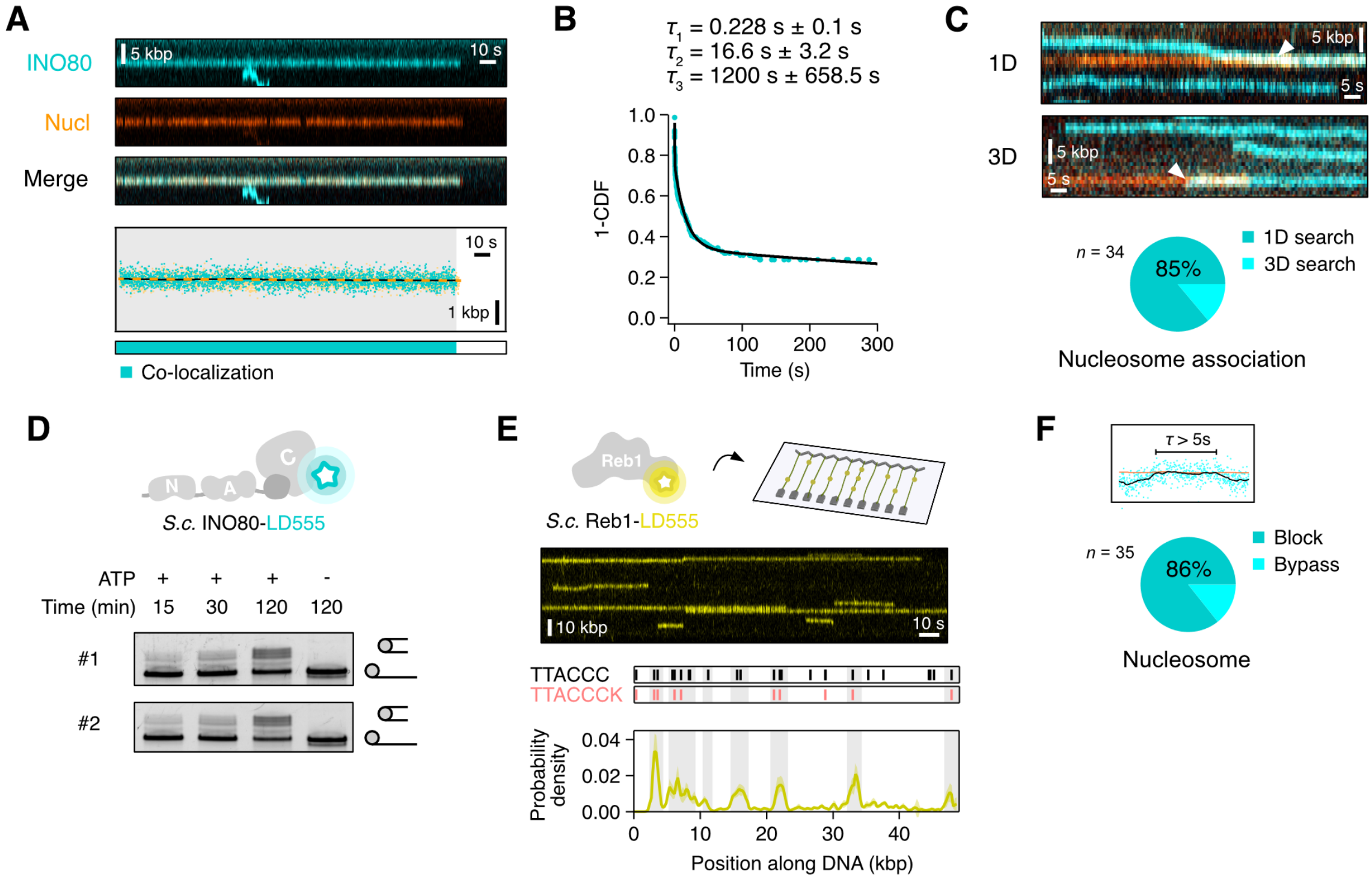

FIG. S6. INO80 interactions with nucleosomes and Reb1. **(A)** Top: Kymogram of an INO80 complex (cyan) co-localizing with a nucleosome (orange). Bottom: Corresponding time traces with co-localizations indicated by shaded areas. **(B)** Top: Lifetimes deducted from best fit for Kaplan-Meier curve. Survival plot of INO80-nucleosome co-localization times. Black line represents best fit. **(C)** Top: Kymograms of INO80 complexes (cyan) associating to nucleosomes (orange) through 1D- and 3D-search. Bottom: Pie chart indicating fraction of INO80-nucleosome-association by 1D- or 3D-search. **(D)** Sliding assays with INO80-LD555 and 0N80 *H.sapiens* nucleosomes. Two representative replicates shown. **(E)** Top: Schematic of Reb1 and DNA curtains assay. Middle: Kymogram of Reb1 binding to λ-DNA. Bottom: Reb1-binding motifs (stronger binding motifs in red) and probability density of Reb1 along λ-DNA. Grey shading indicates peaks of Reb1 binding that correspond with Reb1-binding motifs. Data are presented as mean values +/- 68 % intervals from bootstrapping. n = 724 molecules. **(F)** Pie chart indicating fraction of INO80-nucleosome co-localization events with a length of over 5 s that result in a block. Source data are provided as a Source Data file.

III. DNA SEQUENCES

Nucleosome constructs for gel-based sliding assays contained the following sequences:

601
5’ CTGGAGAATCCCGGTGCCGAGGCCGCTCAATTGGTCGTAGCAAGCTCTAGCACCGC
TTAAACGCACGTACGCGCTGTCCCCCGCGTTTTAACCGCCAAGGGGATTACTCCCTAG
TCTCCAGGCACGTGTCAGATATATACATCCTGT 3’

No A-tract
5’ 601-TGCATGTATTGAACAGCGACCTTGCCTACCACCGTGTACTCGTTGCTCGATCCG
ATCGTTCGTTGCGTAGCTGGCAGTCG 3’

Single
5’ 601-TGCATGTATTGAACAGCGACCTTGCCTACCACCGTGTACTCGTTGCTCGATCCG
TTTTTTTTTTGCGTAGCTGGCAGTCG 3’

Phased
5’ 601-TGCATGTATTGAACAGCGACCTTGCCTACCACCGTGTAAATTTTGCTCAATTTT
CGCGAAATTTGCGTAGCTGGCAGTCG 3’

Phased control
5’ 601-TGCATGTATTGAACAGCGACCTTGCCTACCACCGTGTATATATAGCTCATATATC
GCGATATATGCGTAGCTGGCAGTCG 3’

IV. SUPPLEMENTARY TABLES

TABLE S1. Oligonucleotides (5' to 3') for cloning of protein constructs.

Primer	Sequence
SpyC003-1 fvd	GTAACCACCTTATCAGGTTTATCAGGT
SpyC003-1 rev	AGTATGAGCGTCACCTTCAGTTGC
SpyC003-2 fvd	ACCTGATAAGGTGGTTACACAGCCGCCGCCCATGGTATA
SpyC003-2 rev	GAAGGTGACGCTCATACTCACCACCACCACCACCACTAGTAACTCGA GAGCCCGAAAGGAAGCTGAG
pET28adCas9-1 fvd	CTGCCTTGTGGCAGCTTCAATAACTCGAGTAAGATCCGGC
pET28adCas9-1 rev	GCCGGATCTTACTCGAGTTATTGAAGCTGCCACAAGGCAG
pET28adCas9-2 fvd	CTACAAGCGTTACAAGGGTTCTATGGATAAGAAATACTCA ATAGGCTTAGATATCG
pET28adCas9-2 rev	ATTGAGTATTTCTTATCCATAGAACCCTTGTAACGCTTGTAG
Reb1ybbrHis fvd	TATACATATGGGCAGCGGTTCCGGTAG
Reb1ybbrHis rev	GACCTGAAGGAGCCAGCTTAGAGGCGATGAATTC
Reb1backbn fvd	AAATTAAGATCCGGCTGCTAA
Reb1backbn rev	AACCGCTGCCCATATGTATATCTCCTTCTTAAAGT
Reb1gene fvd	TAAGCTGGCTCCTTCAGGTCATAACGAT
Reb1gene rev	AGCCGGATCTTAATTTTCTGTTTTCATTGAAATATTG
yIno80ybbr fvd	AGAGAGTCGCTTCCCTTGTCGTCATCATCCTTGTAATCG
yIno80ybbr rev	AAGAGAAAATTCAACACTAGACACGG
yIno80pLIB fvd	ACGACAAGGGAAGCGACTCTCTGGAATTCATCGCTTCTAAAC
yIno80pLIB rev	TGTCTAGTGTTGAATTTTCTCTTCATGCTTCGGACCGGGATC

TABLE S2. Oligonucleotides (5' to 3') for cloning of sliding assay constructs.

Primer	Sequence
0-N-80 fvd	CTGGAGAATCCCGGTGCCGAGG
NoAtract-long rev	CGACTGCCAGCTACGCAACGAACGATCGGATCGAGCAACGAG TACACGGTGGTAGGCAAGGTCGCTGTTCAATACATGC
NoAtract-short rev	CGACTGCCAGCTACGCAACGA
Single-long rev	CGACTGCCAGCTACGCAAAAAAAAAACGGATCGAGCAACGA GTACACGGTGGTAGGCAAGGTCGCTGTTCAATACATGC
Single-short rev	CGACTGCCAGCTACGCAAAAAAAAAACG
PhasedATract-long rev	CGACTGCCAGCTACGCAAATTTCGCGAAAATTGAGCA AAATTTACACGGTGGTAGGCAAGGTCGCTGTTCAATACATGC
PhasedATract-short rev	CGACTGCCAGCTACGCAAATTTCGC
PhasedATrContr-long rev	CGACTGCCAGCTACGCATATATCGCGATATATGAGC TATATATACACGGTGGTAGGCAAGGTCGCTGTTCAATACATGC
PhasedATrContr-short rev	CGACTGCCAGCTACGCATATATCGC

[1] L. Cuculis, Z. Abil, H. Zhao, and C. M. Schroeder, TALE proteins search DNA using a rotationally decoupled mechanism, Nat Chem Biol **12**, 831 (2016).

[2] A. B. Kochaniak, S. Habuchi, J. J. Loparo, D. J. Chang, K. A. Cimprich, J. C. Walter, and A. M. Van Oijen, Proliferating Cell Nuclear Antigen Uses Two Distinct Modes to Move along DNA, Journal of Biological Chemistry **284**, 17700 (2009).

[3] P. C. Blainey, G. Luo, S. C. Kou, W. F. Mangel, G. L. Verdine, B. Bagchi, and X. S. Xie, Nonspecifically bound proteins spin while diffusing along DNA, Nat Struct Mol Biol **16**, 1224 (2009).

[4] M. Slutsky and L. A. Mirny, Kinetics of Protein-DNA Interaction: Facilitated Target Location in Sequence-Dependent Potential, Biophysical Journal **87**, 4021 (2004).

[5] K. Kamagata, E. Mano, K. Ouchi, S. Kanbayashi, and R. C. Johnson, High Free-Energy Barrier of 1D Diffusion Along DNA by Architectural DNA-Binding Proteins, Journal of Molecular Biology **430**, 655 (2018).

[6] E. L. Kaplan and P. Meier, Nonparametric Estimation from Incomplete Observations, Journal of the American Statistical Association **53**, 457 (1958).

[7] A. Basu, D. G. Bobrovnikov, Z. Qureshi, T. Kayikcioglu, T. T. M. Ngo, A. Ranjan, S. Eustermann, B. Cieza, M. T. Morgan, M. Hejna, H. T. Rube, K.-P. Hopfner, C. Wolberger, J. S. Song, and T. Ha, Measuring DNA mechanics on the genome scale, Nature **589**, 462 (2021).
