# Supplementary material for: Single-molecule imaging reveals DNA shape read-out by the INO80 chromatin remodeler
Source: Nat Commun. 2026 Sep 29;17:10320. doi: 10.1038/s41467-026-77906-1 (PMC13623949; doi:10.1038/s41467-026-77906-1)
Supplement: Supplementary file 2 [file 41467_2026_77906_MOESM2_ESM.pdf]

Description of Additional Supplementary Files

Supplementary Data 1: Contains raw trajectory data.
