# Supplementary material for: Single-molecule imaging reveals DNA shape read-out by the INO80 chromatin remodeler
Source: Nat Commun. 2026 Sep 29;17:10320. doi: 10.1038/s41467-026-77906-1 (PMC13623949; doi:10.1038/s41467-026-77906-1)
Supplement: Supplementary file 4 [file 41467_2026_77906_MOESM5_ESM.pdf]

nature portfolio

Peer Review File

Single-Molecule Imaging Reveals DNA Shape Read-out by the INO80 Chromatin Remodeler

Corresponding Author: Professor Johannes Stigler

This file contains all reviewer reports in order by version, followed by all author rebuttals in order by version.

Version 0:

Reviewer comments:

Reviewer #1

(Remarks to the Author)
- What are the noteworthy results?
INO80 sliding is DNA shape dependent. INO80 is not observed to, per se, remodel nucleosomes. Nucleosomes and other factors serve as roadblocks to INO80.

- Will the work be of significance to the field and related fields? How does it compare to the established literature? If the work is not original, please provide relevant references.

The work is definitely original. It would be of very high significance to the field as it is a thorough biophysical study of INO80 translocation and interaction along DNA. IT addresses a deficiency in the field. The work is thorough and comprehensive.

- Does the work support the conclusions and claims, or is additional evidence needed?
Mostly yes. Important clarifications need to be made. See comments below.

- Are there any flaws in the data analysis, interpretation and conclusions? - Do these prohibit publication or require revision?
Again, while I do not think there are flaws, clarifications need to be made. See below.

- Is the methodology sound? Does the work meet the expected standards in your field?
Yes. Yes.

- Is there enough detail provided in the methods for the work to be reproduced?
See below.

Comments:

Fig. 1E: not immediately clear what amplitude means.

Salt dependent/ independent: I want to understand why these two concentrations of KGlu were chosen? All the authors should be able to say is that over their explored [KGlu], diffusion coefficient is independent of salt.

“To get insights 113 into INO80’s diffusion mode we analyzed the free-energy landscape along the protein’s path of diffusion by calculating its roughness ε, which causes retardation of the effective diffusion speed due 115 to protein interactions with the DNA”
– I can’t understand what this means. Could the authors please clarify:
(1) what does analysing the free energy landscape along the proteins path of diffusion mean?
(2) What is roughness?
(3) Does ‘which’ in ‘which causes retardation’ refer to ‘roughness’?
(4) The subsequent calculations for roughness and what its predictions should be for linear vs rotational diffusion need to be explained to the unfamiliar reader (such as myself, perhaps as a supplementary note).
(5) Does the author’s conclusion on line 126, stating that INO80 bends DNA in the process, stem only from the fact that Nhp6 has sequence similarity to Nhp10 and that the former has been shown to bend DNA? If so, this conclusion has nothing to do

with roughness or free energy calculation, and so should be moved to a different paragraph (just a thought – feel free to ignore!)

INO80 transfer between strands:
I understood the logic and Fig. S2A-B, but struggled with Fig. S2C-D. When it comes to panel 'C', the authors use am HMM to predict/ identify the time at which inter-strand jump occurs, but there are no details of the HMM model. This of course needs to be addressed – as a reader, I should find enough details in the manuscript to exactly reproduce what the authors found. Then I struggled with this sentence: "To increase accuracy the displacements before and after the identified timepoints were included for analysis and compared to random three-frame displacements" – if the timepoint has already been identified, how is accuracy being increased? What is random three-frame displacement? Also, exactly how is Delta x calculated? This feeds into panel D. In D, how was the data zero centred (am guessing zero is defined as the x and y value at the transfer timepoint but not sure. My glaring deficiency is not understanding what random displacement means. Also, could the authors please clarify if I am correct in assuming that the message here is that for actual jumps, there is significant Delta X without a corresponding Delta Y, and for random displacement, DeltaX accompanies deltaY? Final statement: the reader needs to be 100% crystal clear on whether these jumps are real transfer between strand, and not the same DNA strand waving in the x direction. If you just visualize a single DNA strand (with say dye labelling), what is typical DeltaX? Is that a lot less than some of the DeltaX you observe when the enzyme is present? (I am not suggesting the authors do this experiment, but this is one way I would totally understand).

Shape features:
Since the authors provide pearson's r in the supplementary, the reader will have the expectation of wanting to see what the full scatter distribution (plotted as a heatmap of 2D histogram) would look like. I am also confused as to why the authors do the quintile sorting analysis? Shouldn't a full scatter plot (plotted as a heatmap rather than points) be the most clear way of presenting the data (at least it should be presented somewhere).
Also, are the various other shape parameters, like EL, Stretch, ProT, opening, etc also correlated among themselves, in which case it would be no surprise that if binding parameters correlate with proT, they also correlate with, say, EP.
Further, there are hidden method details that have not been explained in the main text or the supplementary. How is the average propeller twist encountered calculated? The authors must have some rules for determining where along the lambda DNA sequence the protein binds, and where it diassociates, based on the amount of extension of the tether and the distance from the end. The precise rules need to be clarified so that anyone else can exactly reproduce the plots. Right now, this is impossible.
Shape readout is central to the message that the authors was to convey. It becomes important to determine if a similar dependence of diffusion coefficient on region of binding can be explained on the basis of simpler sequence features like AT content. At the very least, the authors should provide a supplementary where they plot a full scatter of D vs mean AT content in the region of the lambda DNA where the trajectory resides and compare its r value to what is reported in fig. S2G. Could they also repeat the same analysis in Fig. 2F, but this time by sorting the data according to quintiles of mean AT content?
Sorry for continuing to play devil's advocate. I do not disbelieve the data, but just was to make really sure that the effect is real and not an artifact of sorting, binning, etc. The authors look at individual trajectories and producing two numbers per trajectory – D and mean PT. But individual trajectories can easily be 4-5 kbp in length (Fig. 1C). So is it true that there are real variations in ProT along lambda DNA over the 4-5 kbp length scale, or over that length scale would ProT variations simply average out? What if I were to chop lambda DNA into 4 kbp fragments – would the mean ProT value of these fragments really be significantly different from each other? Of course I invite counterarguments from the authors regarding whether 4-5 kbp is the right choice or not. If not, then over a typical trajectory, INO80 has already sampled a broad range of ProT values, and in that case, the read dependence comes from trajectories that are much shorter in length.

A-module:
Line 229: the data does not confirm. It is consistent with a hypothesis that the A-module confers the binding preferences to INO80. The data actually show that the A-module retains the same binding preferences as does the INO80 complex, so its highly plausible, but not a 'confirmation' that it is indeed the A-module conferring these properties to INO80.
Lines 235-237: This is inferred. We showed in the paper referenced (21) that linker with low intrinsic cyclisability inhibits INO80 sliding. We showed in a later paper (PMID 36471057) that ProT correlates with intrinsic cyclisability. Hence I believe the authors are inferring what they say, but this should be clarified.

Gel-based assay in Fig 4: beautiful gels!

Fig S5B: The details of a Kaplan meyer curve should be explained in a supplementary note (just a thought – feel free to ignore!)

Overall comment: A very thorough biophysical investigation of how INO80 interacts with DNA, nucleosomes, and other factors. There is enough experimental work done to merit publication, subject to the authors clarifying the points above in detail.

Aakash Basu
Durham University.

Reviewer #2

(Remarks to the Author)
Summary:
This study uses single-molecule DNA curtain imaging to uncover how the chromatin remodeler INO80 locates and positions nucleosomes at promoters and origins of replication. The authors show that INO80 performs facilitated diffusion along DNA via rotationally coupled 1D sliding and intersegmental transfers, allowing it to efficiently search crowded chromatin. Crucially, INO80 preferentially binds DNA regions with specific shape features, especially high negative propeller twist, characteristic of promoter poly(dA:dT) tracts, leading to slower diffusion, longer residence times, and accumulation at these regulatory regions. They identify the conserved A-module of INO80 as the primary DNA shape sensor and demonstrate that recognition of these features can directly inhibit or pause nucleosome sliding, providing a mechanism for precise nucleosome positioning. Additionally, nucleosomes and the barrier protein Reb1 act as passive roadblocks that confine INO80 within promoter regions rather than serving as stable binding partners. Together, the findings reveal how INO80 integrates DNA shape read-out, molecular architecture, and diffusion dynamics to accurately target and remodel nucleosomes, thereby shaping promoter chromatin organization. Over, this is an outstanding study, the experiments are elegant and complimented with detailed quantitative analysis, the manuscript is well written and provides important new mechanistic information on the mechanistic properties of INO80. I have only minor comments and question.

Minor Comments:
In the main text, for figure 1B, please clarify whether ATP was used in the reaction buffer. Are there any difference for plus and minus ATP? Or with non-hydrolyzable ATP analogs?

It would be good to provide a clearer definition of “jumping” versus “intersegmental transfer” based upon the classical definitions of these mechanisms. My understanding of intersegmental transfer is the protein or protein complex in question must have an intermediate state in which it is bound simultaneously to both DNA molecules (or DNA segments). Is there enough structural information available for INO80 to support (or refute) this type of binding mechanism? I assume the answer is “yes”, based upon the models in Figure 6 and the text, but it might be good for readers if this is stated earlier while describing the experimental findings.

One prediction of an intersegmental transfer mechanism is that the frequency of intersegmental transfer should increase in proportion to DNA concentration. If you agree, can this be tested by injecting, for example, plasmid DNA into the flow chamber and asking whether there is enhanced dissociation of the INO80 from the lambda DNA? If so, you may be able to delineate the potential rate of intersegmental transfer in crowded DNA environments (with the understanding that you likely cannot mimic the crowding that occurs in the nucleus). This does not necessarily need to be done for a revision, but it could be an interesting next step in the study.

Shape readout: The data shown in figure 2G tracks with the AT-content of the DNA (i.e. higher AT-content leads to higher propellor twist). It might be useful for readers to highlight the fact that the asymmetric AT-content of the lambda DNA is what allows one to make this measurement.

Reviewer #3

(Remarks to the Author)
In this manuscript, Linder et al used DNA curtains combined with single-molecule imaging to investigate the interaction of the INO80 chromatin remodeling complex with DNA, nucleosomes, and the barrier factor Reb1. They found that INO80 slides along DNA using a rotationally coupled mechanism, and it recognizes DNA shape features that are enriched in promoters and replication origins via the A-module near its N-terminus. INO80 performs 1D searches and intersegmental transfers to dynamically interact with nucleosomes and Reb1. And both nucleosomes and Reb1 block INO80 passage and confine its movement on the DNA. Intriguingly, under their experimental conditions, INO80 does not appear to significantly affect H2A.Z/H2B dimer eviction. Together, these findings reveal how DNA shape, chromatin factors, and molecular architecture can influence INO80’s interaction with DNA and its remodeling activity.
This study provided direct observations in real-time on the interaction between INO80 and DNA, and examined the impact of DNA shape, nucleosome, and Reb1 on its kinetics. The results provided plausible mechanistic views for INO80 promoter targeting and nucleosome remodeling functions. The manuscript can be further strengthened if the authors can test their conclusions or interpretations by manipulating poly(dA:dT)-tracts in the DNA sequence used for DNA curtains.

Version 1:

Reviewer comments:

Reviewer #1

(Remarks to the Author)
The authors had addressed all my concerns satisfactorily. I appreciate the detailed explanations.

Reviewer #2

(Remarks to the Author)
The authors have thoroughly addressed all of the concerns that I raised during the initial review. The revised manuscript is substantially improved, and I am satisfied that the authors have adequately responded to the requested revisions. The study presents technically sound and well-executed work, the methodology is appropriate and described in sufficient detail to support reproducibility, and the data support the authors' conclusions. I have no remaining concerns regarding the analysis, interpretation, or presentation of the results. In my opinion, this work will be of interest to researchers in the field and represents a valuable contribution to the existing literature. I believe the manuscript is appropriate for publication in Nature Communications in its current form.

Reviewer #3

(Remarks to the Author)
The authors addressed my previous question using a gel-based sliding assay. Their new results further support the conclusion that poly(dA:dT)-tracts inhibit INO80 mobility on DNA through DNA shape effects. I have no further comments or questions.

Open Access This Peer Review File is licensed under a Creative Commons Attribution 4.0 International License, which permits use, sharing, adaptation, distribution and reproduction in any medium or format, as long as you give appropriate credit to the original author(s) and the source, provide a link to the Creative Commons license, and indicate if changes were made.
In cases where reviewers are anonymous, credit should be given to 'Anonymous Referee' and the source.
The images or other third party material in this Peer Review File are included in the article's Creative Commons license, unless indicated otherwise in a credit line to the material. If material is not included in the article's Creative Commons license and your intended use is not permitted by statutory regulation or exceeds the permitted use, you will need to obtain permission directly from the copyright holder.
To view a copy of this license, visit https://creativecommons.org/licenses/by/4.0/

Response to reviewer comments

Garp Linder, Daniela Horky, Sarah Zernia and Johannes Stigler

Reviewer 1

- *What are the noteworthy results?*
  INO80 sliding is DNA shape dependent. INO80 is not observed to, per se, remodel nucleosomes. Nucleosomes and other factors serve as roadblocks to INO80.
- *Will the work be of significance to the field and related fields? How does it compare to the established literature? If the work is not original, please provide relevant references.*
  The work is definitely original. It would be of very high significance to the field as it is a thorough biophysical study of INO80 translocation and interaction along DNA. IT addresses a deficiency in the field. The work is thorough and comprehensive.
- *Does the work support the conclusions and claims, or is additional evidence needed?*
  Mostly yes. Important clarifications need to be made. See comments below.
- *Are there any flaws in the data analysis, interpretation and conclusions? - Do these prohibit publication or require revision?*
  Again, while I do not think there are flaws, clarifications need to be made. See below.
- *Is the methodology sound? Does the work meet the expected standards in your field?*
  Yes. Yes.
- *Is there enough detail provided in the methods for the work to be reproduced?*
  See below.

Response
We would like to thank the reviewer for their thoughtful and thorough assessment of our manuscript.

1.1 Fig. 1E: not immediately clear what amplitude means.

Response
To clarify, we changed axis labels in fig. 1 and fig. S1 from 'amplitude' to 'fluorescence (arb. units)' and in the corresponding figure captions from 'amplitude' to 'fluorescence intensity'.

1.2 Salt dependent/ independent:
I want to understand why these two concentrations of KGlu were chosen? All the authors should be able to say is that over their explored [KGlu], diffusion coefficient is independent of salt.

Response
We chose salt concentrations according to the following criteria: the salt concentrations should cover a wide range to observe changes in diffusion speed, but should not disrupt the structural integrity of the complex or the DNA binding ability of both the A- and N-module. In earlier experiments, we observed

that the ΔN-INO80 complex did not bind DNA at ionic strengths above ≈160 mM. Hence, we chose a concentration of 120 mM as a feasible upper limit. We changed the phrasing in the main text:

> This implies that INO80 movement is mostly salt-independent and therefore largely based on sliding over the tested ionic strengths under our experimental conditions.

1.3 Free energy landscapes:
"To get insights into INO80's diffusion mode we analyzed the free-energy landscape along the protein's path of diffusion by calculating its roughness epsilon, which causes retardation of the effective diffusion speed due to protein interactions with the DNA"
– I can't understand what this means. Could the authors please clarify:
(1) what does analysing the free energy landscape along the proteins path of diffusion mean?
(2) What is roughness?
(3) Does 'which' in 'which causes retardation' refer to 'roughness'?
(4) The subsequent calculations for roughness and what its predictions should be for linear vs rotational diffusion need to be explained to the unfamiliar reader (such as myself, perhaps as a supplementary note).
(5) Does the author's conclusion on line 126, stating that INO80 bends DNA in the process, stem only from the fact that Nhp6 has sequence similarity to Nhp10 and that the former has been shown to bend DNA? If so, this conclusion has nothing to do with roughness or free energy calculation, and so should be moved to a different paragraph (just a thought – feel free to ignore!)

Response
Regarding the reviewer's points (1) to (4): To clarify, we added a cartoon figure (shown below as fig. R1) to fig. 2B and added a supplemental note. Note that we now use the term "free-energy barrier" instead of "free-energy roughness", because it was used in previous literature discussing similar topics [1–4]. Additionally, we updated the concerning section in the main text.

Text added as Supplemental Note 1:

> The diffusive motion of a protein along DNA in solution is retarded by two factors: the friction it experiences while moving through the solution and the interactions between DNA and protein. The amount of friction the protein experiences depends on its size, as measured by its radius R. Depending on the nature of its DNA-binding, a protein can diffuse by moving linearly over the DNA employing a rotation-decoupled mechanism [5, 6] or by tracking along the helical axis in a rotational fashion [3]. The diffusion coefficient D scales with $1/R$ for a protein that moves linearly along the DNA and with $1/R^3$ if it additionally rotates along the DNA helix. The second retarding factor is the sum of the protein's interactions with the DNA. To move, it must constantly form and break bonds with DNA, giving rise to free energy barriers along the pathway of diffusion. The height of these energy barriers depends on the characteristics of the protein-DNA contacts and is further modified by the DNA sequence and shape. The resulting retardation is represented by the term $F(\varepsilon) = e^{-\left(\frac{\varepsilon}{k_B T}\right)^2}$, where ε denotes the root-mean-squared variation of the different free-energy barriers that the protein encountered during diffusion (fig. 2B). Altogether, the diffusive motion of the protein can then be described by:
>
> $$D = \frac{k_B T}{\gamma_{lin} + \gamma_{rot}} F(\varepsilon) = \frac{k_B T}{6\pi\eta R + \left(\frac{2\pi}{b}\right)^2 (8\pi\eta R^3 + 6\pi\eta R R_{OC}^2)} e^{-\left(\frac{\varepsilon}{k_B T}\right)^2} \quad \text{(R1)}$$
>
> where γ_{lin} and γ_{rot} represent linear and rotational drag, η is the solvent viscosity, b is the distance between base pairs and R_{OC} is the distance of the protein from the DNA [3]. For proteins moving linearly along DNA γ_{rot} is zero. Models predict ε values between 0.5 to 2 $k_B T$ for efficient target site search [7], which has been confirmed for a wide variety of DBPs [3, 4]. INO80 could hypothetically move along the DNA via a linear or a rotationally coupled mechanism. By measuring D and estimating R as well as R_{OC} from cryo-EM structures, we calculate ε for both possibilities and compare which value is in agreement with the models. Additionally, as explained above, the ε value can give insights into the nature of protein-DNA contacts that occur during binding. Indeed, it has been shown that proteins that induce large conformational changes into the DNA upon binding, e.g., strong bending of the helical axis, exhibit high ε values between 1.4 to 2 $k_B T$.

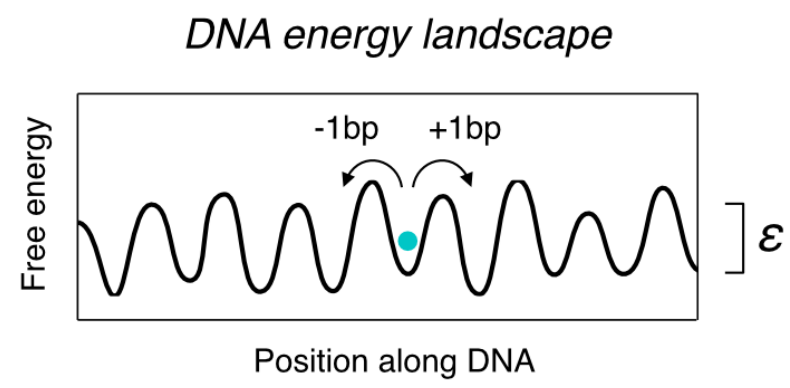

Figure R1: *This subfigure has replaced subfigure fig. 2B.* Example energy landscape felt by a protein sliding along DNA. Energy minima represent binding in register to a particular basepair, while maxima represent the transition state of sliding from one basepair register to the next, i.e., the free-energy barrier for this specific transition. Due to a heterogeneous base sequence, the free energy and barrier heights vary from position to position. As a whole, they can be represented by the root-mean-squared variation ε.

Concerning point (5) of the reviewer's question: Our hypothesis that at least one DNA-binding domain of INO80 bends DNA is also based on the free-energy barrier ε itself. ε of $>1.4\,k_{\mathrm{B}}T$ have been associated with large conformational DNA changes upon binding of the corresponding proteins, e.g. strong bending of the DNA helical axis [4]. In addition to their sequence-similarity both INO80 (Nhp10) and Nhp6 also show comparable ε (INO80: $\approx 1.75\,k_{\mathrm{B}}T$; Nhp6: $\approx 1.71\,k_{\mathrm{B}}T$). To clarify the connection between high ε and DNA bending, we revised the main text and added this aspect to the supplementary note (see above).

1.4 INO80 transfer between strands:
(1) I understood the logic and Fig. S2A-B, but struggled with Fig. S2C-D. When it comes to panel 'C', the authors use am HMM to predict/ identify the time at which inter-strand jump occurs, but there are no details of the HMM model. This of course needs to be addressed – as a reader, I should find enough details in the manuscript to exactly reproduce what the authors found.

Response
In our original analysis, to avoid relying on visual identification, we used an HMM implemented via a single run of the Forward-Backward algorithm to quantify the frames where Inter-DNA switches occur within a trajectory (fig. R2B,C,E). While this analysis largely agreed with visually identified switch positions, the original HMM analysis made the simplifying assumption that the tracked INO80 positions are Markovian, i.e., that their positions in a given frame depend only on their positions in the previous frame. However, for our experimental conditions, this Markovian condition is not strictly met, as the relaxation time of the DNA along x (measured using dCas9 markers to be $\approx$68 ms) is, in fact, not faster than the frame time (50 ms, see fig. R2F).

Prompted by the reviewer's question, we now abandoned the Markovian assumption and re-analyzed our data using an autoregressive hidden Markov model (arHMM), previously implemented for state-hopping in optical-tweezers data [8]. In contrast to a classic HMM, an arHMM can handle time-correlated data, such as position data of INO80 complexes bound to fluctuating DNA substrates. We implemented arHMM by maximizing the trajectory likelihood, produced by the Viterbi algorithm [9, 10], while optimizing the transition rate matrix, DNA fluctuation amplitude, and the state positions. In line with observations by others [8], arHMM produced fewer spurious transitions than our original HMM implementation.

In the manuscript, we replaced the corresponding figures with results from the more accurate arHMM and adjusted the main text. We also now describe the procedure clearly in the methods:

> Inter-DNA switches were determined by an autoregressive Hidden-Markov model (arHMM), previously implemented for identifying state switches in optical tweezer trajectories [8]. For each INO80 movement trajectory perpendicular to the direction of tethering, the likeliest average trajectory (i.e., jumping between neighboring DNAs) was determined by maximizing the trajectory likelihood, provided by the Viterbi algorithm [9, 10], while optimizing the switching probability matrix, DNA fluctuation amplitude, and the state positions, using a More-Hebdon optimizer in Igor Pro 8. The fluctuation dynamics of tethered DNA molecules perpendicular to their tethering direction were determined by tracking DNA-bound dCas9 molecules and analyzing their mean-square displacement relaxation behavior (fig. S2F). MSD fits to the relaxation model

$f(t) = f_\infty + (f_0 - f_\infty)\exp(-t/\tau_r)$ resulted in a transversal relaxation time of ≈68 ms, which was used as an input to the arHMM, and a transversal fluctuation amplitude of ≈139 nm.

Because the arHMM analysis can only detect jumps within a trajectory, it can neither catch long-range jumps in *trans* (between DNA), nor jumps in *cis* (same DNA) (fig. R2A,C). This is because our tracking algorithm separates localizations before and after the jump into independent trajectories if the changes in x and y are too large. To address this, we now identified putative INO80 long-range jumps by collecting all DNA-association events (trajectory starts) within a 200 ms window after each dissociation event (trajectory end). After removing false positives (distinct INO80 complexes that, by chance, associate with a DNA within the 200 ms window), the collected displacements in x and y of identified jumps and displacements arHMM were then combined in a scatter plot (fig. R2A). The addition of long-range jumps now enables a clear distinction between jumps and transfers: jumps are isotropically scattered around the origin, following a near-Gaussian distribution, while transfers form two tight clusters in positive and negative x-direction around the origin (fig. S2G, right graph).

(2) Then I struggled with this sentence: “To increase accuracy the displacements before and after the identified timepoints were included for analysis and compared to random three-frame displacements” – if the timepoint has already been identified, how is accuracy being increased? What is random three-frame displacement? Also, exactly how is Delta x calculated?

Response

To avoid confusion, we abandoned the three-frame displacement procedure. The arHMM produces a list of switch frames and we now plot, for each identified switch occurring between frames $i-1$ and i, the INO80 displacement in x and y (fig. R2G,H). To be precise, we plot $\Delta x = x_i - x_{i-1}$ and $\Delta y = y_i - y_{i-1}$.

(3) This feeds into panel D. In D, how was the data zero centered (I am guessing zero is defined as the x and y value at the transfer timepoint but not sure. My glaring deficiency is not understanding what random displacement means. Also, could the authors please clarify if I am correct in assuming that the message here is that for actual jumps, there is significant Delta X without a corresponding Delta Y, and for random displacement, DeltaX accompanies deltaY?

Response

The origins of our scatter plot and histograms represent the position of INO80 before a jump/transfer, while the data points represent the relative position after the jump/transfer (fig. R2G,H). “Random displacements” included all displacements within trajectories, regardless of whether they were identified as switches or not. These random displacements are hence dominated by the fluctuations of the DNA in x, and the DNA fluctuations and 1D diffusion along the DNA in y. The reviewer is therefore correct in their conclusion that for switches, we observe a substantial change in x without an accompanying change in y. In contrast, in DNA-bound, non-switching situations (1D Diffusion, see fig. R2H), x and y displacements are uncorrelated and, on average, zero. We changed the label from “random displacements” into “1D diffusion”.

(4) Final statement: the reader needs to be 100 % crystal clear on whether these jumps are real transfer between strand, and not the same DNA strand waving in the x direction. If you just visualize a single DNA strand (with say dye labelling), what is typical DeltaX? Is that a lot less than some of the DeltaX you observe when the enzyme is present? (I am not suggesting the authors do this experiment, but this is one way I would totally understand).

Response

Intersegmental transfers are easily identified visually as sudden, but *persistent* changes of the protein’s average position (x-direction) perpendicular to the direction of DNA tethering (y-direction) (fig. R2B,C,E, fig. S4C). As mentioned in (1), we conducted additional experiments and used DNA-bound dCas9 reporters to measure DNA dynamics over time. The DNA molecules indeed fluctuate in the x-direction, with an amplitude of ≈139 nm and a relaxation time of ≈68 ms (determined by tracking DNA-bound dCas9). The separation of transfers and DNA fluctuations is therefore at the timescale level: DNA fluctuations occur on the tens-of-millisecond scale, while transfers occur on the second scale (see fig. R2B,C,E, fig. S4C). Equally importantly, we observed intersegmental transfers between neighboring DNAs only for full-size INO80, with multiple DNA-binding domains, but not for the A-module mutant of INO80 or dCas9.

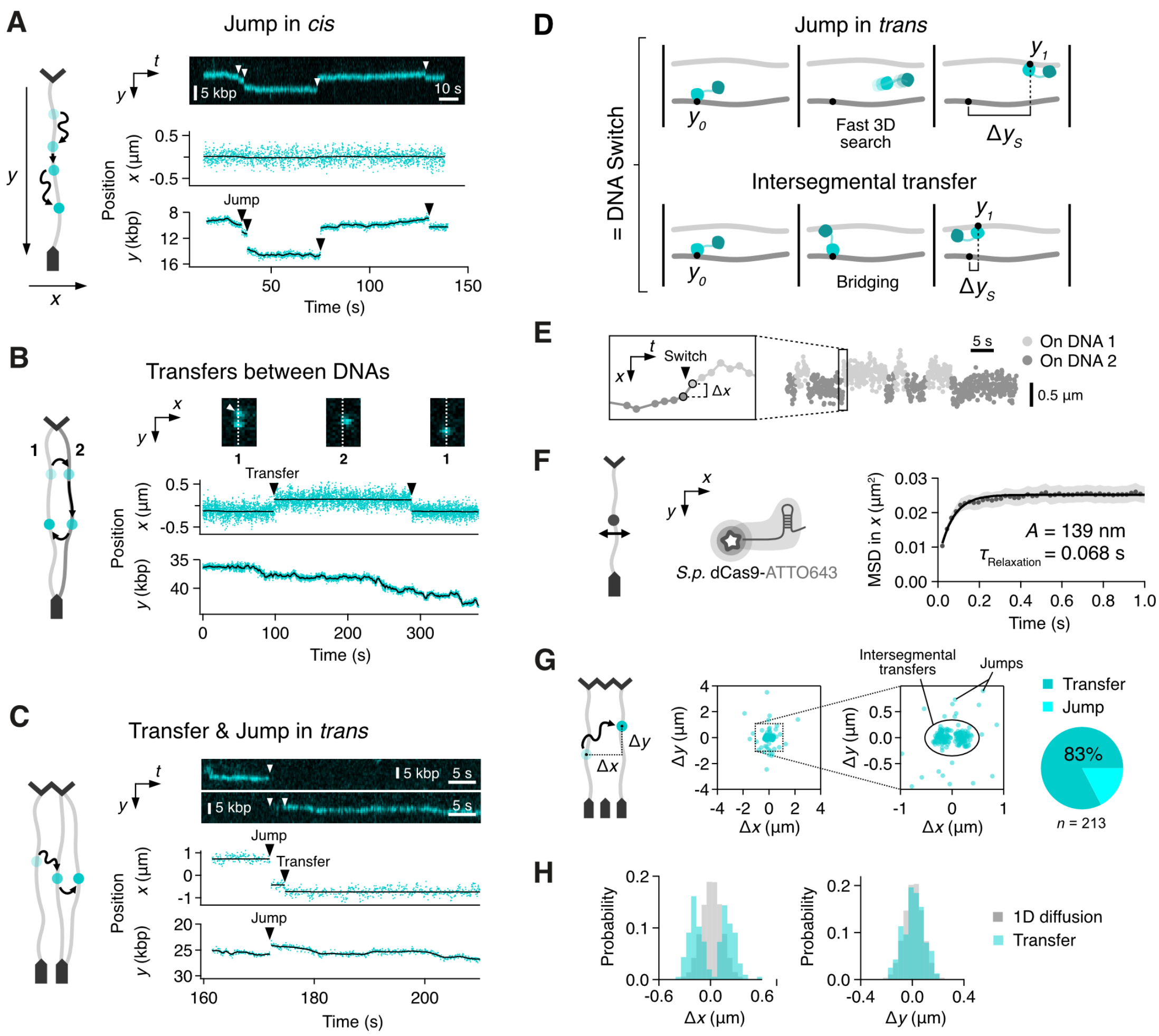

Figure R2: *This figure has been added to the manuscript as fig. S2.* **(A)** Example of four long-distance jumps of a single INO80 complex along the same DNA strand. Left: Schematic of the jumping events on the same DNA. Right top: Kymogram of the trajectory. Triangles indicate jumps. Right bottom: x- and y-position of the complex over time. **(B)** Example of two transfer events by a single INO80 complex. Left: Schematic of the transfer events from DNA (1) to (2) to (1). Right top: TIRF microscopy images of the complex before and after each transfer event (striped line represents position of DNA (1)). White triangles indicates relevant INO80 complex. Right bottom: x- and y-position of the complex over time. **(C)** Example of a jumping event between two DNAs followed by a transfer event. Rest as fig. S2A. **(D)** Schematic of DNA switch mechanisms structurally available to the INO80 complex. Switching using "jumps" entails complete dissociation of all DNA-binding domains and an intermediate 3D search; "intersegmental transfers" are performed through a bridging intermediate in which each DNA is bound by a different DNA-binding interface of the complex. Because 3D search is several magnitudes faster than 1D search and the bridging distance is limited by the size of the protein, jumps are, on average, accompanied by a larger instantaneous change in y- or x-position. **(E)** Example x-trajectory of an INO80 complex. Approximate switch timeframes were identified using an arHMM, and localizations were assigned to one of two DNAs. **(F)** Schematic depicting transversal movement of the λ-DNA in x, measured using a dCas9-marker protein. Right: Average MSD in x-direction of dCas9-markers and relaxation time of the transversal DNA-movement, $n = 21$. **(G)** Left: Schematic depicting a jump of an INO80 molecule and the according x- and y-displacements. Middle: Displacements in x and y for inter-DNA switches and jumps identified either by an arHMM or inter-trajectory jump analysis. Right: Middle plot zoomed in towards the origin, $n = 213$. **(H)** Analysis of intersegmental transfers. Displacements within the two clusters (cyan) were compared to INO80 displacements during 1D search (grey). Left: Histogram of x-displacements. Right: Histogram of y-displacements, $n_{\mathrm{Transfer}} = 176$.

1.5 Shape features:

(1) Since the authors provide pearson's r in the supplementary, the reader will have the expectation of wanting to see what the full scatter distribution (plotted as a heatmap of 2D histogram) would look like. I am also confused as to why the authors do the quintile sorting analysis? Shouldn't a full scatter plot (plotted as a heatmap rather than points) be the most clear way of presenting the data (at least it should be presented somewhere).

Response

We added the full scatter plots to the supplementary information, similarly to their depiction in fig. 2F, fig. 3E and fig. S4B,D (fig. R3).

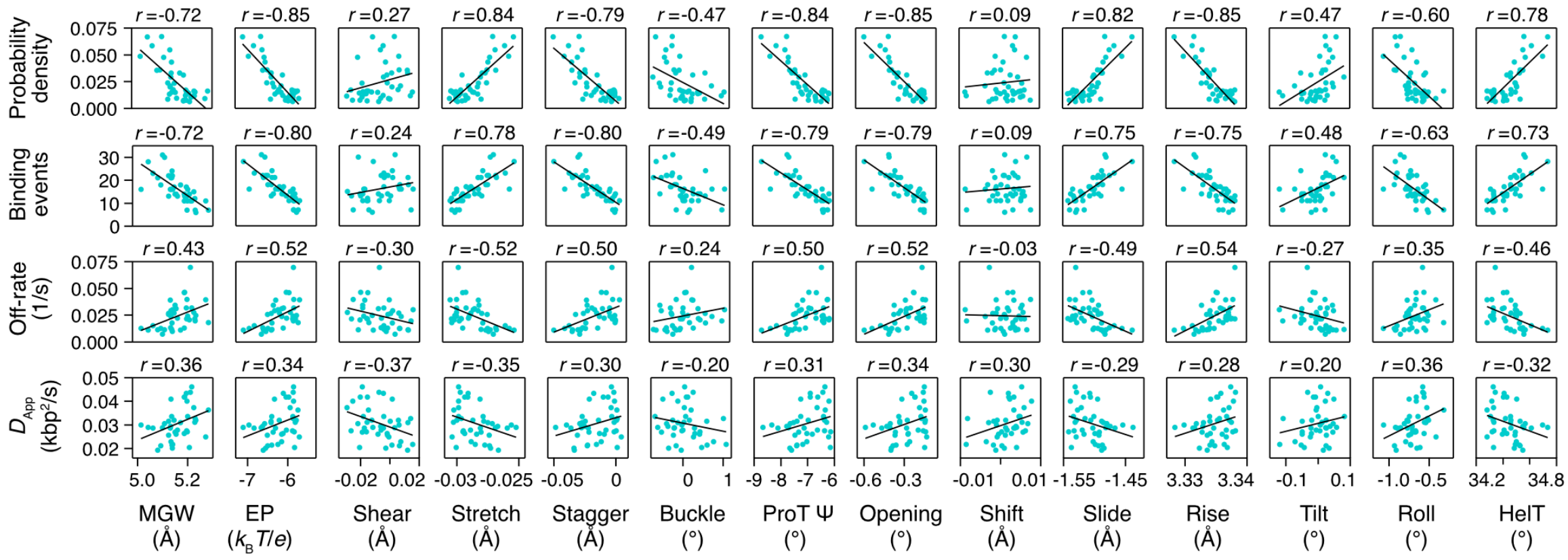

Figure R3: *This figure has been added to the manuscript as fig. S3D.* Correlation plots and Pearson's correlation coefficients of different DNA shape features with DNA binding parameters of INO80 along λ-DNA shown in fig. S3B.

Plotting the data as heatmaps is unfeasible because we are inherently limited by the number of bins. Because this was also a point of confusion in (3) and (5), we would like to briefly clarify how we determine the position of a molecule on the DNA, why binning is necessary and why we chose 1 kbp-bins.

We calculate the location of a particle on the λ-DNA in relation to the positions of the chromium anchors and barriers, to which the DNA is tethered. While DNAs are all tethered in the same orientation, their tethering points on the bottom pedestals may differ. This adds to the localization error of the microscope when mapping molecules to DNA, thereby limiting overall resolution precision. We calculated an RMSD of ±800 bp. Our selection of 1 kbp bins was chosen because it exceeds this limit and provides a sensible balance between having many small vs few large bins. We emphasize that the detected effects are also visible in different binning (fig. R4A).

We acknowledge that the presentation and calculation of the quintile analysis were rather unclear, so we removed it. We now instead use the position-dependent apparent diffusion coefficient D_{App} already established in fig. 3 and fig. S4 (fig. R4B). D_{App} is calculated using the displacements measured within a chosen short timeframe Δt. Contrary to the diffusion coefficient values calculated for the whole trajectory of a respective molecule, D_{App} can then be binned according to the position of the respective displacements, like the other position-dependent observables, and therefore does not suffer from averaging or length biases. We have added the corresponding plots to fig. 2F. The findings are similar to those of the quintile analysis, showing a slight correlation between higher diffusion speeds and lower $|\Psi|$ values (fig. R4B). This fits our observations that the diffusion speed in the wild-type complex is mainly determined by the high free-energy barrier of Nhp10's HMG-boxes, which bind non-sequence-specifically. Consistent with this, removing the N-module increased the shape dependence on diffusion speed drastically (fig. 3B,E).

(2) Also, are the various other shape parameters, like EL, Stretch, ProT, opening, etc also correlated among themselves, in which case it would be no surprise that if binding parameters correlate with proT, they also correlate with, say, EP.

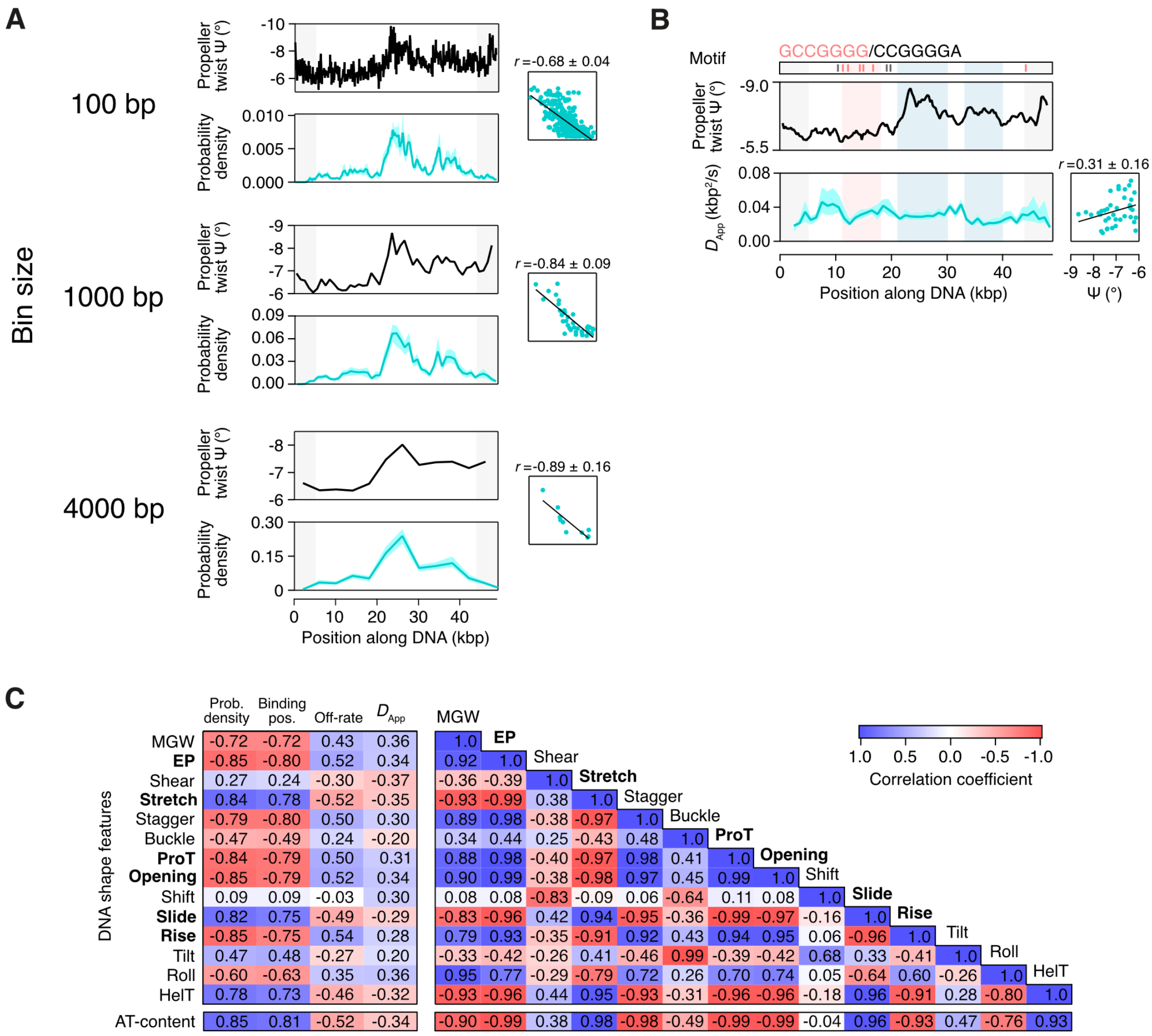

Figure R4: **(A)** *This plot has been added to the manuscript as fig. 3A.* Effect of bin size on correlations of INO80 probability density with mean propeller twist values across the λ-DNA. **(B)** *This plot has been added to subfigure fig. 2F.* Nhp10-Motifs, average propeller twist (1 kbp sliding window) and D_{App} along λ-DNA. Shaded areas: close to chrome barriers with unreliable data (grey), accumulation of Nhp10 motifs (red), and high negative propeller twist (blue). Grey shaded areas were not included in the correlation analysis. $n = 677$. Right box: correlation of binding parameters with average propeller twist and Pearson's correlation coefficient. **(C)** *This plot has replaced subfigure fig. S2H.* Pearson's correlation coefficients for correlation of different DNA shape features and AT-content with DNA binding parameters of INO80 along λ-DNA shown in fig. 2F and for correlation among themselves.

Response

Indeed, most shape parameters are correlated, because they all (besides EP) merely represent a different geometric aspect of the same 3D structure. The electrostatic potential within the minor groove itself is included because it is directly dependent on the DNA shape [11] and is a physical quantity, which is directly recognized by many DNA-binding proteins [12, 13]. To quantify the connections between AT content and shape parameters, as well as between shape parameters themselves, we correlated the feature values predicted for the DNA used in the experiments across 1 kbp bins. Indeed, all features that correlated highly with INO80's binding patterns (shown in bold) also correlate extremely well with each other ($r > 0.94$, fig. R4C).

(3) Further, there are hidden method details that have not been explained in the main text or the supplementary. How is the average propeller twist encountered calculated? The authors must have some rules for determining where along the lambda DNA sequence the protein binds, and where it dissociates, based on the amount of extension of the tether and the distance from the end. The precise rules need to be clarified so that anyone else can exactly reproduce the plots. Right now, this is impossible.

Response

Localizations were mapped to DNA positions as explained in (1). We added an explanation of how the positions of the molecule on the λ-DNA are determined, and how the propeller twist is calculated, to the methods section.

(4) Shape readout is central to the message that the authors was to convey. It becomes important to determine if a similar dependence of diffusion coefficient on region of binding can be explained on the basis of simpler sequence features like AT content. At the very least, the authors should provide a supplementary where they plot a full scatter of D vs mean AT content in the region of the lambda DNA where the trajectory resides and compare its r value to what is reported in fig. S2G. Could they also repeat the same analysis in Fig. 2F, but this time by sorting the data according to quintiles of mean AT content?

Response

The reviewer addresses an important question: Could INO80's behavior on DNA be explained by AT content rather than DNA shape?

As discussed in (2), DNA features that correlate strongly with INO80's binding patterns (shown in bold) also correlate extremely well with each other ($r > 0.94$, fig. R4C). EP, ProT and AT-content especially show near-perfect correlation ($r > 0.99$), making it impossible for us to distinguish their contributions at the resolution of our fluorescence microscopy experiments. This is now also mentioned in the main text.

To distinguish the relevance of DNA shape features vs. high-AT content in a functional context, we performed sliding assays, which allowed us to test our shape read-out hypothesis during remodeling. We already showed that a single long A-tract reduced remodeling speed, but the effect was rather small (fig. R5A,C,D). Prompted by the large DNA-binding interface of the A-module and the distribution of A-tracts in *S. cerevisiae* promoters [14], we introduced three short A-tracts in succession, phased by 10 bp (fig. R5A), so that they face the same side. Strikingly, the insertion of phased A-tracts into the linker DNA almost abrogated INO80 sliding (fig. R5C,D). To test whether this inhibition originated through recognition of shape or just AT-content, we exchanged the A-tracts with $(AT)_n$-sequences, which have identical AT content but lower $|\Psi|$ (fig. R5A, fig. S5A). This led to a significant speed-up of the remodeling process, although the sliding remained slightly slower than for the single A-tract substrate. These results indicate that recognition of DNA shape, rather than AT content, at multiple positions/interfaces of the A-module is necessary for inhibition of sliding activity.

Prompted by the sliding assay results, we further asked whether our results are relevant *in vivo*. Thus, we added an analysis of shape profiles around *in vivo* +1 nucleosome positions, mapped by H3Q85C chemical cleavage published in [15]. To account for the heterogeneity of promoter structures in yeast, we sorted the +1 nucleosomes into subclasses defined in [16] and additionally analyzed promoters with or without a canonical TATA-box (fig. R5E). We found a strong high propeller twist signal over ≈25 bp within the NDR of constitutive and insulated as well as TATA-less *S. cerevisiae* promoters, which corresponds positionally to the N-terminal end of the A-module, if INO80 is bound to the +1 nucleosome (fig. R5F, fig. S5D,E). If recognition of this high propeller twist 'signal' inhibits remodeling, as it does in our sliding assays, this would prevent INO80 from sliding nucleosomes into the NDR and thereby establish

positioned nucleosomes. We further show that this signal is fixed to the position of the +1 nucleosome dyad and not the midpoint of the neighboring NDR (fig. R5G). Finally, analysis of uni- and bidirectional promoters shows that the signal is weaker for −1 nucleosomes (fig. R5H). We now discuss these new insights in the main text.

(5) Sorry for continuing to play devil's advocate. I do not disbelieve the data, but just was to make really sure that the effect is real and not an artifact of sorting, binning, etc. The authors look at individual trajectories and producing two numbers per trajectory – D and mean PT. But individual trajectories can easily be 4-5 kbp in length (Fig. 1C). So is it true that there are real variations in ProT along lambda DNA over the 4-5 kbp length scale, or over that length scale would ProT variations simply average out? What if I were to chop lambda DNA into 4 kbp fragments – would the mean ProT value of these fragments really be significantly different from each other? Of course I invite counterarguments from the authors regarding whether 4-5 kbp is the right choice or not. If not, then over a typical trajectory, INO80 has already sampled a broad range of ProT values, and in that case, the read dependence comes from trajectories that are much shorter in length.

Response
Yes, the λ-DNA does show variations over 4 to 5 kbp (see fig. R4A), and our conclusions still hold at different levels of binning, which is now also mentioned in the main text. Please refer to our response to point (1).

1.6 A-module:
(1) Line 229: the data does not confirm. It is consistent with a hypothesis that the A-module confers the binding preferences to INO80. The data actually show that the A-module retains the same binding preferences as does the INO80 complex, so its highly plausible, but not a 'confirmation' that it is indeed the A-module conferring these properties to INO80.

Response
Following the reviewer's advice, we have changed the wording in the conclusion:

> Taken together, our data is consistent with the model that the A-module acts as a DNA shape sensor that confers strong binding preferences for DNA with high $|\Psi|$ to the INO80 complex.

---

(2) Lines 235-237: This is inferred. We showed in the paper referenced (21) that linker with low intrinsic cyclisability inhibits INO80 sliding. We showed in a later paper (PMID 36471057) that ProT correlates with intrinsic cyclisability. Hence I believe the authors are inferring what they say, but this should be clarified.

Response
The reviewer is correct. We therefore added a DNA shape analysis of the linker sequences used in (fig. R6). We indeed found that all rigid linkers showed considerably higher $|\Psi|$ values. We added the results of the analysis to the supplement (fig. S5C) and changed the paragraph in the main text:

> These results indicate that, rather than AT-content, the A-module specifically recognizes sequences with high $|\Psi|$ or EP. In agreement with this finding, a previous study had found reduced remodeling speeds for the nucleosomes with native rigid linker sequences [17], which showed higher $|\Psi|$ than their flexible counterparts (fig. S5C).

1.7 Fig S5B: The details of a Kaplan meyer curve should be explained in a supplementary note (just a thought – feel free to ignore!)

Response
We have added a supplementary note:

> In fig. S1D, fig. 5D and fig. S6B we plot INO80 lifetime and nucleosome interaction data in form of a survival curve using the Kaplan–Meier method [18]. In our case, it shows the cumulative

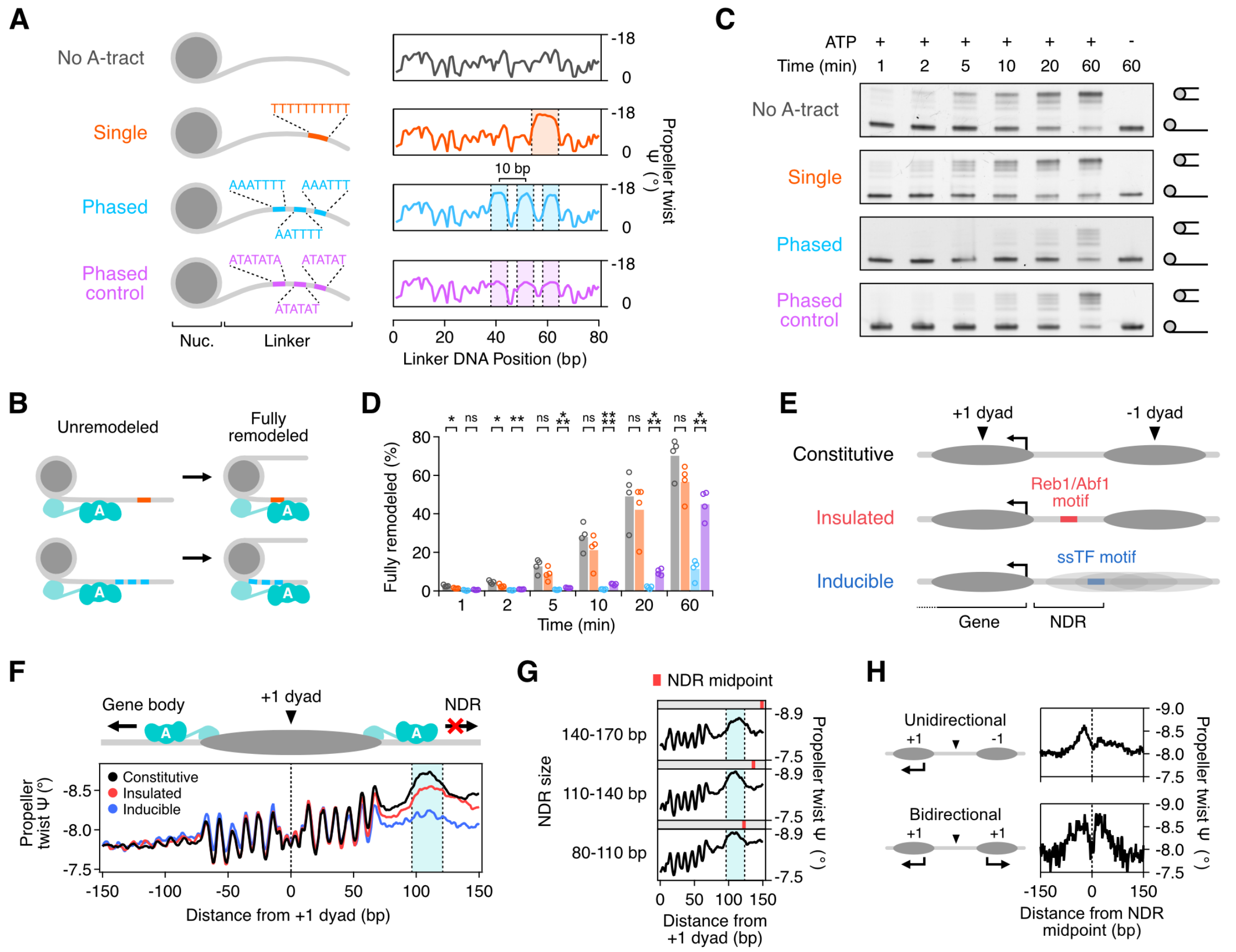

Figure R5: *This figure replaces the old fig. 4* Effect of DNA shape sensing on nucleosome remodeling. **(A)** Left: Schematic of 0N80 nucleosome design for gel-based sliding assays. Sequences exchanged are shown. Right: Predicted DNA shape parameter for linker DNA of all nucleosome constructs. Areas highlighted indicate changes in shape compared to the control sequence. **(B)** Native gel showing remodeling of 0N80 nucleosomes over time depending on linker DNA. Bottom band represents unremodeled end-positioned nucleosome, top band represents fully remodeled center-positioned nucleosome. **(C)** Comparison of fully remodeled nucleosome fraction for all nucleosomal substrates. Significance was determined using a two-tailed t-test. $n = 4$. **(D)** Schematic showing the positioning of exchanged sequences in respect to INO80 ATPase (light cyan) and A-module (cyan) before and after remodeling, based on structural data and biochemical mapping. **(E)** Schematic showing the architecture of *S. cerevisiae* promoters classified in ref. [16]. ssTF = sequence-specific transcription factors. **(F)** Bottom: Average shape profiles around +1-nucleosomes of different *S. cerevisiae* promoter classes oriented according to the direction of transcription. The region of high propeller twist is highlighted. $n_{\mathrm{UNB}} = 2390$, $n_{\mathrm{TFO}} = 1737$, $n_{\mathrm{STM}} = 920$ Top: Schematic showing the superimposed positioning of the INO80 ATPase and A-module bound to the +1 nucleosome in the two possible orientations. INO80 pulls nucleosomes in the direction of its bound A-module. Nucleosome positions were determined in ref. [15] by H3Q85C chemical cleavage mapping. **(G)** Average shape profiles around subsets of +1-nucleosomes of *S. cerevisiae* UNB promoters with different NDR sizes, oriented according to the direction of transcription. The region of high propeller twist is highlighted. Average NDR midpoint for each subset is indicated in red. **(H)** Average shape profiles around NDR midpoints for all uni- and bidirectional promoters in *S. cerevisiae*. $n_{\mathrm{Uni}} = 4955$, $n_{\mathrm{Bi}} = 587$.

survival probabilities of fluorophores (fig. S1D) or interactions (fig. 5D and fig. S6B) at time t and is described by:

$$\hat{S}(t) = \prod_{i:t_i \leq t} \left(1 - \frac{d_i}{n_i}\right) \tag{R2}$$

where d_i is the number of bleached fluorophores or dissolved interactions and d_i the number of fluorophores/interactions at risk. It allows inclusion of fluorophores or interactions that did not bleach or dissolve within the observed time interval (censored data) and can be fitted to extract single or multiple lifetimes ($\tau_1, \tau_2 ...$), depending on the complexity of the interaction.

Gel-based assay in Fig 4: beautiful gels!
Overall comment: A very thorough biophysical investigation of how INO80 interacts with DNA, nucleosomes, and other factors. There is enough experimental work done to merit publication, subject to the authors clarifying the points above in detail.

Reviewer 2

Summary:
This study uses single-molecule DNA curtain imaging to uncover how the chromatin remodeler INO80 locates and positions nucleosomes at promoters and origins of replication. The authors show that INO80 performs facilitated diffusion along DNA via rotation-coupled 1D sliding and intersegmental transfers, allowing it to efficiently search crowded chromatin. Crucially, INO80 preferentially binds DNA regions with specific shape features, especially high negative propeller twist, characteristic of promoter poly(dA:dT) tracts, leading to slower diffusion, longer residence times, and accumulation at these regulatory regions. They identify the conserved A-module of INO80 as the primary DNA shape sensor and demonstrate that recognition of these features can directly inhibit or pause nucleosome sliding, providing a mechanism for precise nucleosome positioning. Additionally, nucleosomes and the barrier protein Reb1 act as passive roadblocks that confine INO80 within promoter regions rather than serving as stable binding partners. Together, the findings reveal how INO80 integrates DNA shape read-out, molecular architecture, and diffusion dynamics to accurately target and remodel nucleosomes, thereby shaping promoter chromatin organization. Over, this is an outstanding study, the experiments are elegant and complimented with detailed quantitative analysis, the manuscript is well written and provides important new mechanistic information on the mechanistic properties of INO80. I have only minor comments and question.

Response
We would like to thank the reviewer for their positive evaluation of our work.

2.1 In the main text, for figure 1B, please clarify whether ATP was used in the reaction buffer. Are there any difference for plus and minus ATP? Or with non-hydrolyzable ATP analogs?

Response
Buffers for all experiments contained 1 mM ATP. To assess whether ATP influences INO80 interaction

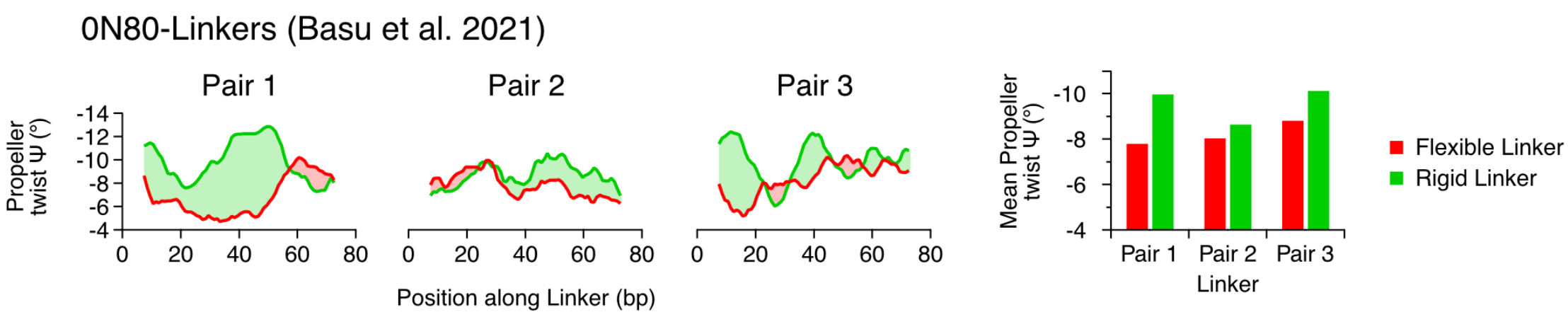

Figure R6: *This figure has been added to the manuscript as fig. S5C.* DNA Shape analysis of 0N80-Linkers used in [17]. Left: Average propeller twist (15 bp sliding window) along the linker sequence for all three linker pairs tested. Right: Average propeller twist for all linker sequences compared.

with DNA, we conducted DNA curtains assays without adding ATP at the same salt concentration and compared the results. Removal of ATP had no impact on INO80 binding preferences (fig. R7A), but molecules showed significantly lower lifetimes and faster diffusion speeds (fig. R7B,C). This suggests that ATP binding may strengthen INO80-DNA interactions.

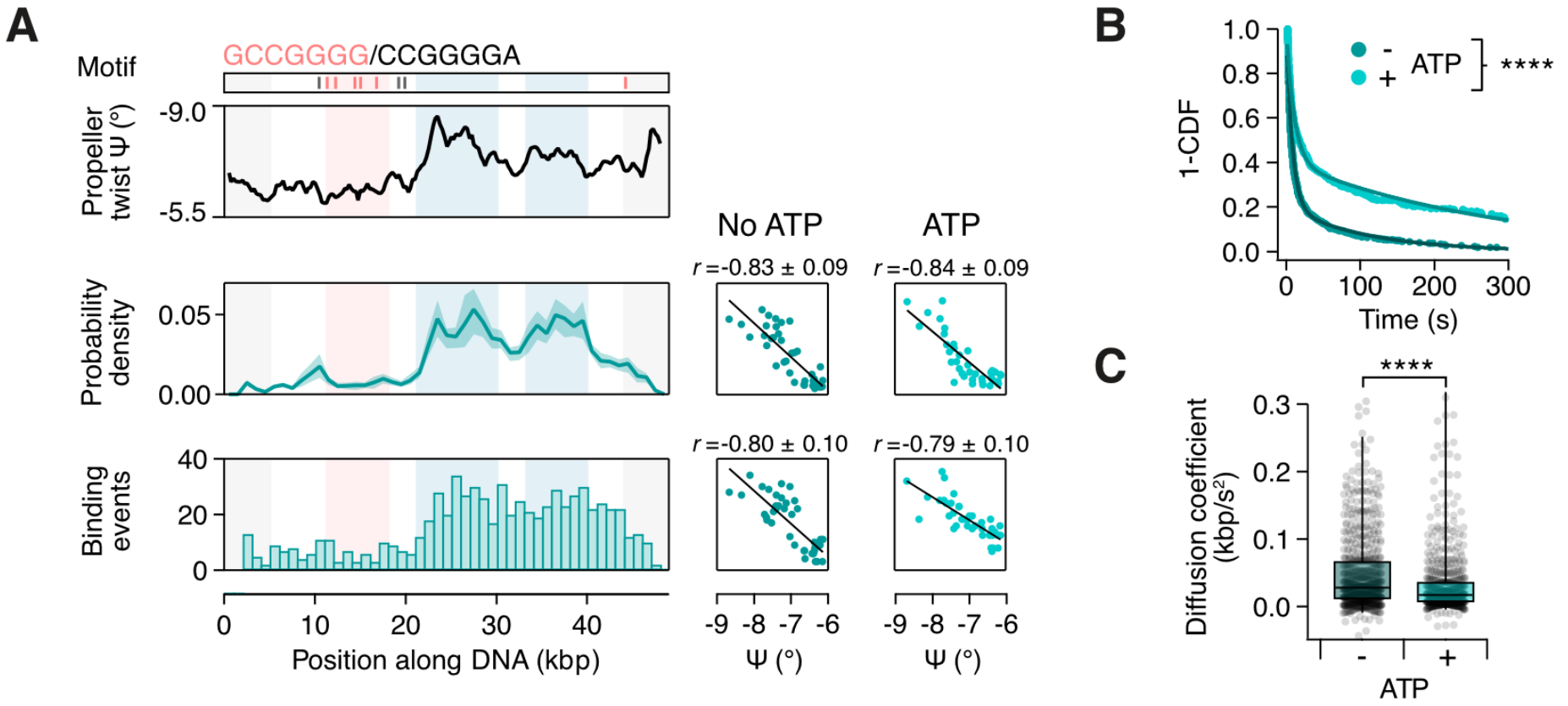

Figure R7: *This figure has been added to the manuscript as fig. 2G–I, fig. S2D.* **(A)** Nhp10-Motifs, average propeller twist (1 kbp sliding window) and different binding parameters along λ-DNA for experiments without ATP. Shaded areas: close to chrome barriers with unreliable data (grey), accumulation of Nhp10 motifs (red), high negative propeller twist (blue). Grey shaded areas were not included in correlation analysis. $n = 719$. Right boxes: correlation of binding parameters with average propeller twist and Pearson's correlation coefficient for experiments with and without ATP in the buffer. **(B)** Survival plot of INO80 on λ-DNA for experiments with and without ATP. Rates were compared with Log-Rank test. **(C)** Comparison of diffusion coefficients of INO80 on λ-DNA for experiments with and without ATP. Significance was determined using a two-tailed Mann-Whitney U test. $n_{\text{noATP}} = 719$, $n_{\text{ATP}} = 677$.

2.2 It would be good to provide a clearer definition of "jumping" versus "intersegmental transfer" based upon the classical definitions of these mechanisms. My understanding of intersegmental transfer is the protein or protein complex in question must have an intermediate state in which it is bound simultaneously to both DNA molecules (or DNA segments). Is there enough structural information available for INO80 to support (or refute) this type of binding mechanism? I assume the answer is "yes", based upon the models in Figure 6 and the text, but it might be good for readers if this is stated earlier while describing the experimental findings.

Response

The terms 'jump', 'hop' and 'transfer' are often used interchangeably in the literature, which can lead to confusion. To make the text more accessible, we now use the term 'jump in *cis*' for jumps on the same DNA, 'switch' for all cases in which INO80 changes DNA strands, and, as subcategories of switches, define both 'jumps in *trans*' and 'intersegmental transfers' more clearly. A new subfigure depicts the molecular mechanisms (see fig. R2D). We now explain the mechanisms in the results part and note that INO80 fulfills all the structural prerequisites for intersegmental transfers. Please also our response to Reviewer 1.4.

2.3 One prediction of an intersegmental transfer mechanism is that the frequency of intersegmental transfer should increase in proportion to DNA concentration. If you agree, can this be tested by injecting, for example, plasmid DNA into the flow chamber and asking whether there is enhanced dissociation of the INO80 from the lambda DNA? If so, you may be able to delineate the potential rate of intersegmental transfer in crowded DNA environments (with the understanding that you likely cannot mimic the crowding that occurs in the nucleus). This does not necessarily need to be done for a revision, but it could be an interesting next step in the study.

Response

We agree with the reviewer that increasing the concentration of accessible DNA should, in principle,

increase the probability of intersegmental transfer. Injecting soluble competitor DNA into the flow chamber would therefore be an informative future experiment to test whether INO80 dissociation from λ-DNA is enhanced under more DNA-crowded conditions.

We have not performed this experiment in the present study and therefore avoid making quantitative claims about DNA-concentration-dependent transfer rates or crowded nuclear environments. Since this point is outside the scope of the present revision and, as the reviewer notes, not essential to the revision, we have not added new experimental data on this point.

2.4 Shape readout: The data shown in figure 2G tracks with the AT-content of the DNA (i.e. higher AT-content leads to higher propellor twist). It might be useful for readers to highlight the fact that the asymmetric AT-content of the lambda DNA is what allows one to make this measurement.

Response
We added this sentence to the main text:

> Bacteriophage λ-DNA shows great variations in propeller twist over long segments (fig. 2F), allowing us to investigate the relationship between INO80 binding parameters and Ψ values.

Reviewer 3

Summary:
In this manuscript, Linder et al used DNA curtains combined with single-molecule imaging to investigate the interaction of the INO80 chromatin remodeling complex with DNA, nucleosomes, and the barrier factor Reb1. They found that INO80 slides along DNA using a rotation-coupled mechanism, and it recognizes DNA shape features that are enriched in promoters and replication origins via the A-module near its N-terminus. INO80 performs 1D searches and intersegmental transfers to dynamically interact with nucleosomes and Reb1. And both nucleosomes and Reb1 block INO80 passage and confine its movement on the DNA. Intriguingly, under their experimental conditions, INO80 does not appear to significantly affect H2A.Z/H2B dimer eviction. Together, these findings reveal how DNA shape, chromatin factors, and molecular architecture can influence INO80's interaction with DNA and its remodeling activity. This study provided direct observations in real-time on the interaction between INO80 and DNA, and examined the impact of DNA shape, nucleosome, and Reb1 on its kinetics. The results provided plausible mechanistic views for INO80 promoter targeting and nucleosome remodeling functions.

3.1 The manuscript can be further strengthened if the authors can test their conclusions or interpretations by manipulating poly(dA:dT)-tracts in the DNA sequence used for DNA curtains.

Response
This would indeed be an interesting experiment. However, our attempts to introduce long A-tract sequences to isolate the influence of AT-content and shape features into the DNA failed, presumably because their inherent repetitiveness led to extensive recombination during cloning. We hence addressed this issue in gel-based sliding assays (see 1.5).

References

[1] Maria Barbi et al. "A Model of Sequence-Dependent Protein Diffusion Along DNA". In: *Journal of Biological Physics* 30.3 (2004), pp. 203–226. DOI: 10.1023/B:JOBP.0000046728.51620.14.

[2] Leonid Mirny et al. "How a Protein Searches for Its Site on DNA: The Mechanism of Facilitated Diffusion". In: *J. Phys. A: Math. Theor.* 42.43 (Oct. 2009), p. 434013. DOI: 10.1088/1751-8113/42/43/434013.

[3] Paul C Blainey et al. "Nonspecifically Bound Proteins Spin While Diffusing along DNA". In: *Nat Struct Mol Biol* 16.12 (Dec. 2009), pp. 1224–1229. DOI: 10.1038/nsmb.1716.

[4] Kiyoto Kamagata et al. "High Free-Energy Barrier of 1D Diffusion Along DNA by Architectural DNA-Binding Proteins". In: *Journal of Molecular Biology* 430.5 (Mar. 2018), pp. 655–667. DOI: 10.1016/j.jmb.2018.01.001.

[5] Luke Cuculis et al. "TALE Proteins Search DNA Using a Rotationally Decoupled Mechanism". In: *Nat Chem Biol* 12.10 (Oct. 2016), pp. 831–837. DOI: 10.1038/nchembio.2152.

[6] Anna B. Kochaniak et al. "Proliferating Cell Nuclear Antigen Uses Two Distinct Modes to Move along DNA". In: *Journal of Biological Chemistry* 284.26 (June 2009), pp. 17700–17710. DOI: 10.1074/jbc.M109.008706.

[7] Michael Slutsky and Leonid A. Mirny. "Kinetics of Protein-DNA Interaction: Facilitated Target Location in Sequence-Dependent Potential". In: *Biophysical Journal* 87.6 (Dec. 2004), pp. 4021–4035. DOI: 10.1529/biophysj.104.050765.

[8] Brian A. Dawes and Maria Kamenetska. "Autoregressive HMM Resolves Biomolecular Transitions from Passive Optical Tweezer Force Measurements". In: *Biophysical Journal* 124.19 (Oct. 2025), pp. 3115–3123. DOI: 10.1016/j.bpj.2024.11.3320.

[9] A. Viterbi. "Error Bounds for Convolutional Codes and an Asymptotically Optimum Decoding Algorithm". In: *IEEE Trans. Inf. Theory* 13.2 (Apr. 1967), pp. 260–269. DOI: 10.1109/TIT.1967.1054010.

[10] William H. Press et al. *Numerical Recipes: The Art of Scientific Computing*. 2007.

[11] Barry Honig and Anthony Nicholls. "Classical Electrostatics in Biology and Chemistry". In: *Science* 268.5214 (May 1995), pp. 1144–1149. DOI: 10.1126/science.7761829.

[12] Remo Rohs et al. "The Role of DNA Shape in Protein–DNA Recognition". In: *Nature* 461.7268 (Oct. 2009), pp. 1248–1253. DOI: 10.1038/nature08473.

[13] Zengqin Deng et al. "Mechanistic Insights into Metal Ion Activation and Operator Recognition by the Ferric Uptake Regulator". In: *Nat Commun* 6.1 (July 2015). DOI: 10.1038/ncomms8642.

[14] Randy Wu and Hao Li. "Positioned and G/C-capped Poly(dA:dT) Tracts Associate with the Centers of Nucleosome-Free Regions in Yeast Promoters". In: *Genome Res.* 20.4 (Apr. 2010), pp. 473–484. DOI: 10.1101/gr.103226.109.

[15] Răzvan V. Chereji et al. "Precise Genome-Wide Mapping of Single Nucleosomes and Linkers in Vivo". In: *Genome Biol* 19.1 (Dec. 2018), p. 19. DOI: 10.1186/s13059-018-1398-0.

[16] Matthew J. Rossi et al. "A High-Resolution Protein Architecture of the Budding Yeast Genome". In: *Nature* 592.7853 (Apr. 2021), pp. 309–314. DOI: 10.1038/s41586-021-03314-8.

[17] Aakash Basu et al. "Measuring DNA Mechanics on the Genome Scale". In: *Nature* 589.7842 (Jan. 2021), pp. 462–467. DOI: 10.1038/s41586-020-03052-3.

[18] E. L. Kaplan and Paul Meier. "Nonparametric Estimation from Incomplete Observations". In: *Journal of the American Statistical Association* 53.282 (June 1958), pp. 457–481. DOI: 10.1080/01621459.1958.10501452.
